# Functional Dendrimer Nanogels for DNA Delivery and Gene Therapy of Tumors

**DOI:** 10.1002/anie.202505669

**Published:** 2025-05-05

**Authors:** Xin Li, Zhijun Ouyang, Laura Hetjens, Ming Ni, Kuailu Lin, Yong Hu, Xiangyang Shi, Andrij Pich

**Affiliations:** ^1^ Institute of Technical and Macromolecular Chemistry RWTH Aachen University 52074 Aachen Germany; ^2^ DWI‐Leibniz‐nstitute for Interactive Materials 52074 Aachen Germany; ^3^ College of Biological Science and Medical Engineering Donghua University Shanghai 201620 China; ^4^ Department of Orthopaedics Rujin Hospital Shanghai Jiao Tong University School of Medicine Shanghai 200025 China; ^5^ Department of Breast Surgery The First Affiliated Hospital of Wenzhou Medical University Wenzhou 325015 China; ^6^ Department of Polymeric Materials School of Materials Science and Engineering Tongji University Shanghai 201804 China; ^7^ Aachen Maastricht Institute for Biobased Materials Maastricht University Geleen 6167 RD The Netherlands

**Keywords:** Dendrimer nanogels, DNA delivery, Emulsion‐free, Multiresponsiveness, Self‐triggered degradation

## Abstract

Solving the dilemma between efficacy and cytotoxicity of cationic colloidal vectors is one of the biggest challenges in gene delivery. Cationic dendrimer assemblies with hierarchical structure, smart and biomimetic behaviors have been developed for drug/gene delivery in vivo. Among different dendrimer assemblies, the dendrimer‐based nanogels were not intensively studied due to complicated synthesis and unknown properties. Here, for the first time, low‐generation dendrimer nanogels with high yield and purity, tunable size, uniform morphology, and good colloidal stability were synthesized using the emulsion‐free method, which cannot be obtained by the miniemulsion method. Importantly, the dendrimer nanogels integrate the advantages of low‐generation dendrimer and stimuli‐responsive polymer, thus achieving dual‐active groups, *o*‐hydroxyl amine units, temperature‐responsiveness, polyampholyte property, and self‐triggered aminolysis. With these unique properties, dendrimer nanogels can “temporarily” acquire high charge density through the covalent crosslinking of low‐generation dendrimer for improved DNA compression, promoted cell internalization and lysosomal escape, and efficient DNA delivery, followed by self‐triggered aminolysis into small dendrimers to control DNA release, reduce cytotoxicity, and facilitate metabolism in vivo. Compared to high‐generation dendrimers, low‐generation dendrimer nanogels display higher gene transfection and therapeutic efficacies, and lower side effects simultaneously. This work provides a facile strategy for the preparation of low‐generation dendrimer nanogels that break up the contradiction between efficacy and cytotoxicity of cationic colloidal vectors in gene therapy. This innovative approach to construct low‐generation dendrimers into smart dendrimer nanogels will have broad applicability in clinical translation.

## Graphical abstract

Dendrimer nanogels are designed by emulsion‐free method, endowing them with dual‐active groups, *o*‐hydroxyl amine, temperature‐responsiveness, polyampholyte property, and self‐triggered aminolysis. With these unique characters, dendrimer nanogels display high gene transfection and therapeutic efficacies with low side effects, thus solving the dilemma between efficacy and cytotoxicity of conventional cationic colloidal vectorsin gene delivery.

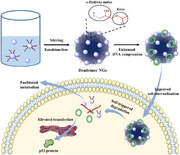

## Introduction

Nanogels (NGs) have attracted tremendous attention in the field of biomedicine (e.g., drug delivery, disease diagnosis and therapy), due to their unique properties like tunable size, high loading efficiency, excellent stability, and stimuli‐responsiveness.^[^ ^1^, ^2^ ^]^ In particular, 3D crosslinked network of NGs enables capacious loading and controlled release of complex macromolecular species, such as proteins and nucleic acids.^[^ ^3^, ^4^, ^5^, ^6^ ^]^ Among them, poly(*N*‐vinylcaprolactam) (PVCL)‐based NGs are conspicuous because of good colloidal stability, superior biocompatibility, and a volume phase transition temperature (VPTT) close to body temperature.^[^ ^7^, ^8^, ^9^ ^]^ Likewise, the stimuli‐responsive characters of PVCL‐based NGs can be tailored using the corresponding functional comonomers and crosslinkers.^[^ ^10^ ^]^

Cationic poly(amido amide) (PAMAM) dendrimers with hyperbranched architecture, well‐defined surface chemistry, and low immunogenicity have also received considerable attention in the biomedical fields.^[^ ^11^, ^12^, ^13^ ^]^ The multivalency of PAMAM dendrimers can facilitate drug loading and gene compacting, tissue penetration, and cellular uptake by active transcytosis.^[^ ^14^, ^15^, ^16^ ^]^ Else, PAMAM dendrimers with p*K*a value of 6.0 and pH buffering ability display excellent endosomal escape behavior.^[^ ^17^ ^]^ Nevertheless, the inherent shortcomings of PAMAM dendrimers, such as ultrasmall size (<10 nm), low loading capacity, imperfect tissue permeability and retention effect, hinder their clinical transformation. To overcome these predicaments, various dendrimer assemblies (e.g., clusters, micelles, or vesicles) have been constructed.^[^ ^18^, ^19^ ^]^ In particular, several studies demonstrated that the fusion of low‐generation dendrimers into assemblies enables efficient drug/gene delivery with ignorable side effects.^[^ ^20^, ^21^ ^]^

**Scheme 1:**
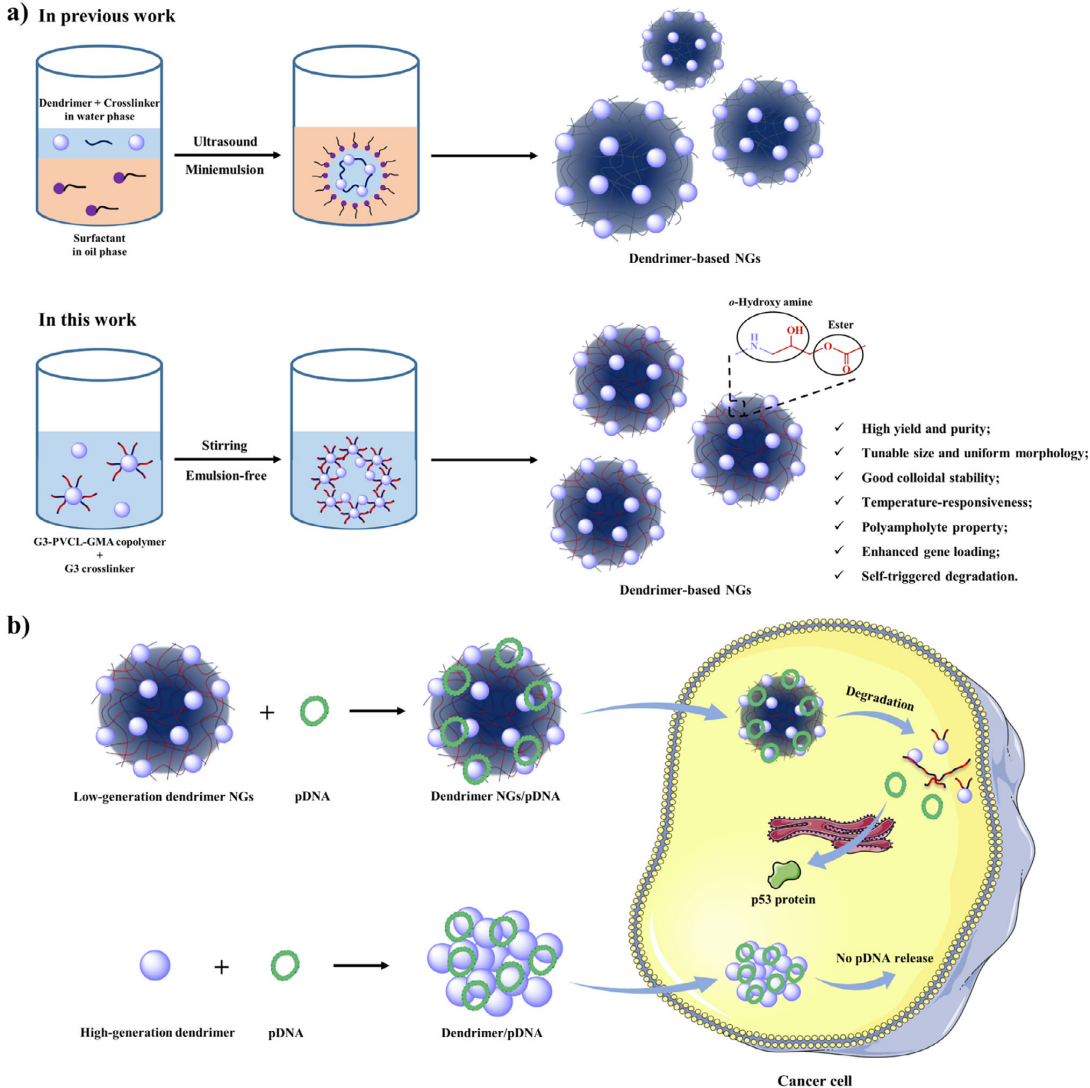
a) Schematic illustration of the synthesis of dendrimer‐based NGs using the miniemulsion (in previous work) or emulsion‐free method (in this work). The dendrimer‐based NGs obtained by emulsion‐free method exhibit several advantages. b) Schematic illustration of dendrimer NGs for highly efficient DNA delivery and gene therapy. Compared to high‐generation dendrimers, low‐generation dendrimer NGs display higher gene therapeutic efficacy and lower side effects simultaneously, due to their unique properties.

Indeed, dendrimer NGs are superior to other dendrimer assemblies in the aspects of colloidal stability, adjustable size and morphology, as well as tailored stimuli‐responsiveness.^[^ ^22^ ^]^ To date, the synthesis of dendrimer NGs mainly depends on the classical miniemulsion method (Scheme 1a).^[^ ^23^, ^24^ ^]^ However, the miniemulsion method exhibits various shortcomings such as the complex operating procedure, hard‐to‐purify attribute, and low NG yield.^[^ ^25^ ^]^ Besides the as‐synthesized dendrimer NGs show nonuniform size distribution, poor colloidal stability, and high cytotoxicity owing to the unideal emulsification process and the difficult removal of surfactants.^[^ ^26^ ^]^

Here, for the first time, we developed a facile emulsion‐free method for the synthesis of dendrimer NGs with high yield and purity, tunable size, uniform morphology, and good colloidal stability (Scheme 1a). PVCL‐GMA copolymer is first synthesized through reversible addition‐fragmentation chain transfer (RAFT) polymerization, and then conjugated to low‐generation G3 dendrimer to obtain amphiphilic G3‐PVCL‐GMA copolymer which is self‐assembled in base buffer solution and further crosslinked by G3 dendrimer to afford G3‐NGs. Through integrating G3 dendrimer and PVCL‐GMA copolymer, the formed G3‐NGs display the unique features of dual‐active groups (amino and epoxy), *o*‐hydroxyl amine units, temperature‐responsiveness, polyampholyte property, and self‐triggered aminolysis. Remarkably, a very recent work discovered that the *o*‐hydroxyl amine unit in polymers obtained by amino‐epoxy polymerization is beneficial to improving gene compression and delivery efficiencies.^[^ ^27^ ^]^

For gene delivery, the transfection efficiency of common NGs is unsatisfactory due to their poor degradability and relatively low charge density.^[^ ^28^, ^29^, ^30^ ^]^ The colloidal vectors with high molecular weight or/and high charge density are essential for gene complex stabilization and delivery, but lead to serious cytotoxicity.^[^ ^31^, ^32^ ^]^ Therefore, breaking down the contradiction between efficacy and cytotoxicity of cationic colloidal vectors is an urgently challenge in gene delivery.^[^ ^33^ ^]^ Encouragingly, in this work, the temperature‐responsiveness and polyampholyte property allow G3‐NGs to exhibit high density of positive charge at body temperature and acidic microenvironment, which is conducive to form compact complexes, protect DNA from nuclease degradation, promote cell internalization and subsequent endosomal escape. The high charge density of G3‐NGs endows efficient DNA delivery, after which they are degraded into small dendrimers by the self‐triggered aminolysis of ester bonds to control DNA release, reduce cytotoxicity, and promote metabolism in vivo. Remarkably, compared to high‐generation dendrimers, low‐generation dendrimer NGs display higher gene therapeutic efficacy and lower side effects simultaneously (Scheme 1b). This work provides a facile strategy for the synthesis of low‐generation dendrimer NGs that break up the efficacy–cytotoxicity correlation of cationic colloidal vectors in gene therapy.

## Results and Discussion

**Figure 1:**
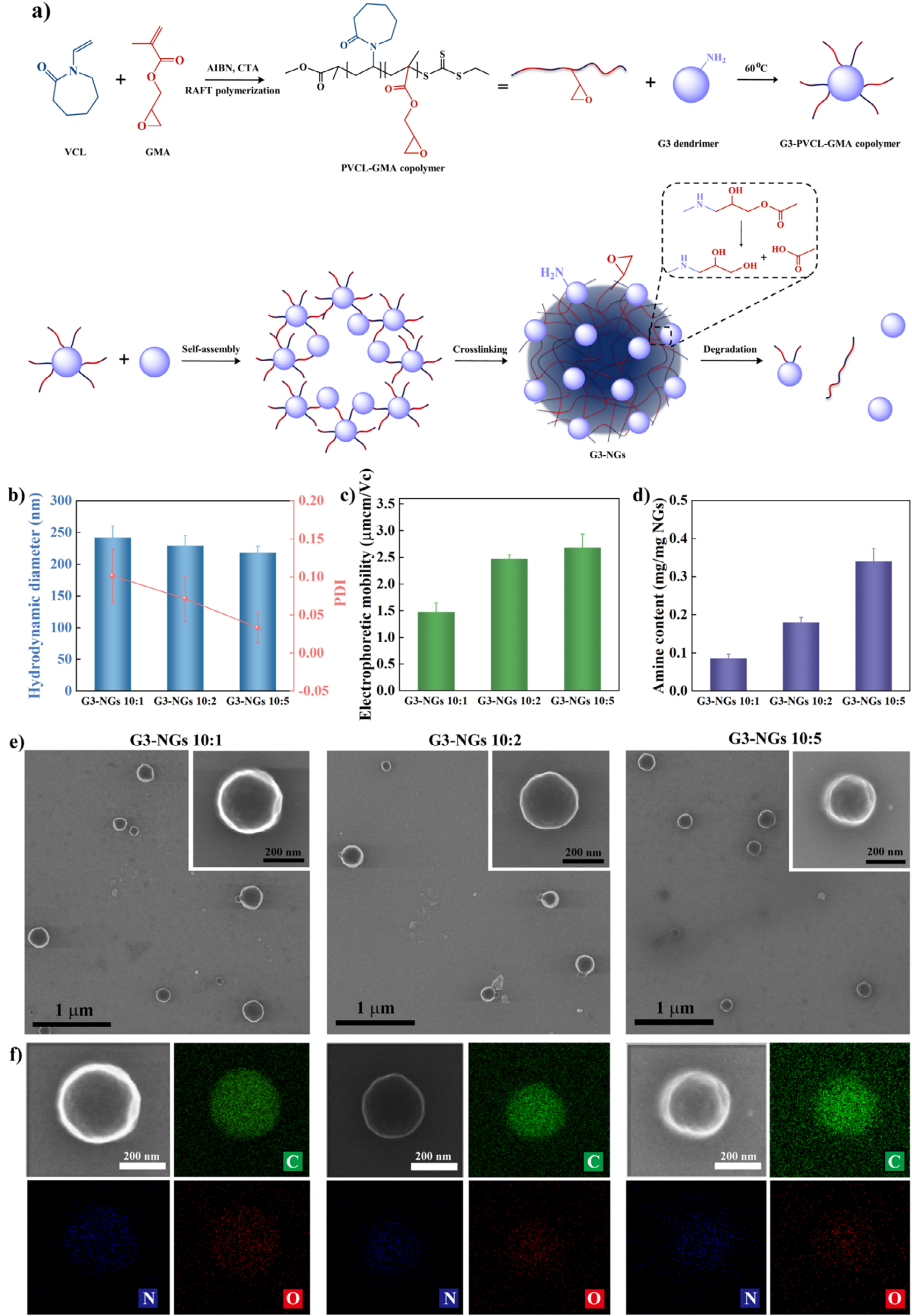
a) Schematic illustration of emulsion‐free synthesis and self‐triggered aminolysis of G3‐NGs. b) Hydrodynamic diameters and the corresponding PDIs, c) electrophoretic mobilities, d) primary amine contents, e) STEM images, and f) the corresponding elemental mapping images of G3‐NGs (*n* = 3 independent experiments).

The monomers of *N*‐vinylcaprolactam (VCL) and glycidyl methacrylate (GMA), the initiator of 2,2′‐azobis(2‐methylpropionitrile) (AIBN), and the chain transfer agent (CTA) of methyl 2‐(ethoxycarbonothioylthio) propanoate were chosen to synthesize PVCL‐GMA copolymer through RAFT polymerization (Figure 1a). The PVCL‐GMA copolymer was characterized (Table S1 and Figures S1–S3), and the result of GPC showed that PVCL‐GMA copolymer exhibits the average molecular weights (*M*_n_) of 5309 and dispersity of (*Đ*) of 1.27. In FTIR spectra, the characteristic peaks at 1622 and 1726 cm^−1^ were from the carbonyl vibration of VCL and GMA, respectively. Meanwhile, based on our previous calculation method,^[^ ^34^ ^]^ the actual GMA content in PVCL‐GMA copolymer was about 16.7 mol% which is higher than the theoretical GMA content of 10 mol%. The obtained PVCL‐GMA copolymer was hydrophobic due to its high GMA content. Furthermore, PVCL‐GMA copolymer was conjugated on low‐generation G3 dendrimer by the reaction of epoxy and amino groups to form the amphiphilic G3‐PVCL‐GMA copolymer that possesses the *M*_n_ of 19 982 and *Đ* of 1.12 (Table S1 and Figures S4–S6). The number of PVCL‐GMA copolymers conjugated on each G3 dendrimer was calculated to be around 3.0.

Next, G3 dendrimer was employed as crosslinker to covalently crosslink the G3‐PVCL‐GMA copolymer to afford low‐generation dendrimer‐based nanogels (G3‐NGs) by the emulsion‐free method (Figures 1a, S7). The obtained solution became a homogeneous turbid dispersion, indicating that polymer colloids is formed (Figure S8). With the content increase of G3 dendrimer, the hydrodynamic diameters of G3‐NGs decreased from about 250 to 210 nm (Figure 1b), while their electrophoretic mobilities increased from around 1.47 to 2.68 µmcm Vc^−1^ (Figure 1c). Likewise, the formed G3‐NGs displayed the narrow size distribution and low PDIs (<0.15) (Figures 1b, S9, and S10). Remarkably, a very high NG yield of more than 90% was achieved using the facile emulsion‐free method (Figure S11), as compared with the low yield (less than 30%) of NG synthesis by the conventional miniemulsion method. Additionally, the uniform morphology and element distribution of G3‐NGs were determined via scanning transmission electron microscopy (STEM) and energy‐dispersive X‐ray spectroscopy (EDX) (Figures 1e,f, S12). With the content increase of G3 dendrimer, the amount of N element gradually increased. This is in line with the increasing trend of primary amine mass in G3‐NGs from 0.085 to 0.34 mg mg^−1^, as determined by the commercial primary amino nitrogen (PANOPA) assay (Figure 1d).

**Figure 2:**
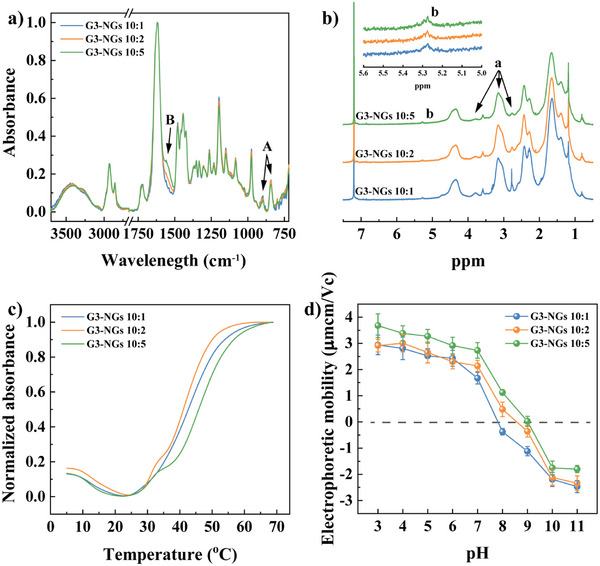
a) FTIR and b) ^1^H NMR spectra of G3‐NGs. c) Cloud point analysis of G3‐NGs at the temperatures of 5–70 °C. d) Electrophoretic mobilities of G3‐NGs at pH 3–11 (*n* = 3 independent experiments).

To confirm the covalent crosslinking within NGs, the FTIR spectra of G3‐NGs were measured (Figure 2a). The oxirane ring asymmetric expansion and contraction vibrations (*v*_GMA_) at 908 and 814 cm^−1^ (Peak A) were the characteristic bands of GMA in the copolymer. The N─H stretching vibration (*v*_N‐H_) at 1562 cm^−1^ (Peak B) was from the crosslinked bonds (─NH─) between G3 dendrimer and copolymer. Clearly, the peak intensities of *v*_GMA_ and *v*_N‐H_ from G3‐NGs were weakened and enhanced with the increase of G3 crosslinker respectively, indicating that the amino groups of G3 dendrimer were successfully crosslinked with the epoxy groups of copolymer. Besides, in the NMR spectra (Figure 2b), the characteristic signals of 2.8, 3.1, and 3.8 ppm (Peak a) were assigned to GMA of copolymer, and the signal of 5.2–5.3 ppm (Peak b) represented the crosslinked bonds (─NH─) from G3‐NGs. With the increase of G3 crosslinker, the signals from GMA were intensified, and the signal from ─NH─ was appeased progressively. These results implied that G3‐NGs with dual‐active groups (amino and epoxy) and *o*‐hydroxyl amine unit can be prepared by the emulsion‐free method. Importantly, the *o*‐hydroxyl amine unit is capable of improving gene compression and delivery efficiencies.^[^ ^27^ ^]^ Likewise, the dual‐active groups can be employed for conjugating versatile functional molecules via facile amine or/and epoxy chemistry, thus endowing NGs with the desired properties in biomedical applications.^[^ ^7^, ^35^ ^]^

The temperature‐responsiveness of G3‐NGs was characterized from the cloud point, dynamic light scattering, and electrophoretic mobility. Interestingly, G3‐NGs exhibited the UCST‐LCST dual‐thermal transition behavior due to the copresence of highly hydrophilic G3 dendrimer and temperature‐responsive PVCL‐based polymer (Figure 2c). Initially, the UCST behavior showed that the light scattering intensity decreases with the temperature increase from 5 to 25 °C. It may attribute to the formation of hydrogen bonds between water molecules and the amino/carbonyl groups of G3 dendrimer, which leads to the swelling of G3‐NGs to increase their transparency (Figure S13). After that, as the temperature increases further, the LCST behavior presented that the light scattering intensity increases (the appearance of turbidity) due to the collapse of PVCL‐based polymer chains. Remarkably, the UCST‐LCST dual‐thermal transition behavior of G3‐NGs is in agreement with that of zwitterionic PVCL‐based NGs.^[^ ^36^ ^]^ Moreover, the UCST‐LCST dual‐thermal transition behavior was also observed in the hydrodynamic diameter change of G3‐NGs (Figure S13). The size increase of G3‐NGs associated with the UCST behavior is limited (only about 5–10 nm). With a further temperature increase, the LCST behavior showed that G3‐NGs shrink to around 130–150 nm. Likewise, the temperature‐dependent electrophoretic mobility of G3‐NGs was tested (Figure S14). With the increase of temperature, the electrophoretic mobility of G3‐NGs was gradually increased. This is attributed to the concentration of charge by the shrinking of NG outer layer. The temperature‐responsive charge concentration property is beneficial for stabilizing nanocarriers, avoiding drug/gene leakage in vivo circulation, promoting cellular internalization, and subsequent endosomal escape.

Additionally, polyampholyte property of G3‐NGs was validated by the electrophoretic mobility measurement (Figure 2d). In G3‐NGs, the amino groups can be protonated in acidic condition and epoxy groups can be hydrolyzed to form hydroxyl functionalities in alkaline condition, that renders G3‐NGs an isoelectric point (IEP) and reversible charge conversion. Because of the different amounts of amino and epoxy groups within G3‐NGs, they displayed different IEP values. The G3‐NGs (10:1) and G3‐NGs (10:2) showed the low IEP of about pH 7.8 and 8.5, respectively. Although the largest IEP of about pH 9.1 in G3‐NGs (10:5) was mainly attributed to the high amount of amino groups, these results indicated that G3‐NGs have polyampholyte property and can reversibly convert the charge from positive to negative by responding to the pH of different microenvironments. Therefore, the polyampholyte property endows G3‐NGs with the capabilities of pH‐responsive controlled drug/gene release and promoted endosomal escape in acidic cancer cells.^[^ ^9^, ^22^ ^]^

**Figure 3:**
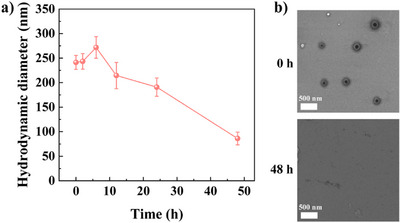
a) Variation of hydrodynamic diameter and b) STEM images of G3‐NGs in pH 6.8 for different degradation times (*n* = 3 independent experiments).

The biodegradability of NGs is a crucial feature for biomedical applications. The amino groups of G3‐NGs can self‐trigger aminolysis of the ester bonds within the NG network.^[^ ^23^ ^]^ Through DLS measurement and STEM image (Figure 3), the aminolysis of G3‐NGs 10:2 was assessed in the simulated tumor microenvironment (pH 6.8). Apparently, G3‐NGs 10:2 were progressively degraded within 48 h. Moreover, the size of G3‐NGs 10:2 increased at 5 h as they become loose and then swell after the partial aminolysis of ester bonds. Subsequently, the size of G3‐NGs 10:2 was continuously decreased until the completed degradation at 48 h. These results demonstrated that the self‐triggered aminolysis of G3‐NGs can be employed to control drug/gene release and facilitate their in vivo metabolism to reduce the toxicity and the risk of long‐term retention.^[^ ^37^ ^]^ However, the aminolysis property of G3‐NGs also leads to the poor stability. Therefore, the storage and use of G3‐NGs should be concerned. G3‐NGs is stored in the form of dry powder, and the NG solution is freshly prepared in subsequent experiments.

**Figure 4:**
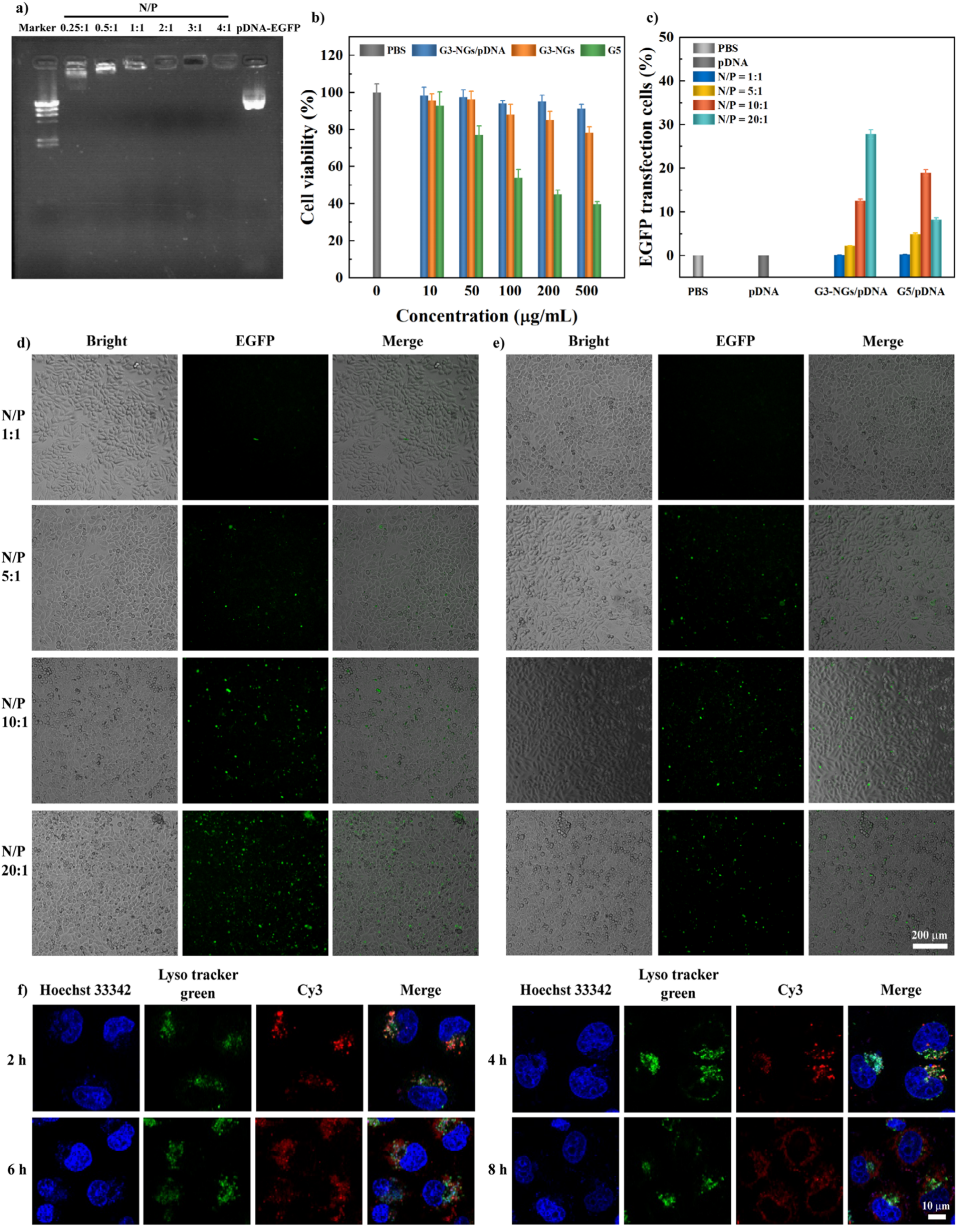
a) Gel retardation assay of G3‐NGs/pDNA polyplexes at different N/P ratios. b) Viability assay of HeLa cells treated with PBS, G3‐NGs, G5, and G3‐NGs/pDNA polyplexes at different concentrations for 24 h (*n* = 3 independent experiments). c) EGFP gene transfection efficacy and d), e) fluorescent microscopy image of HeLa cells in d) G3‐NGs/pDNA and e) G5/pDNA polyplexes at different N/P ratios (*n* = 3 independent experiments). The data of EGFP gene transfection efficacy is from flow cytometry. f) Confocal microscopic images of HeLa cells incubated with G3‐NGs/pDNA polyplexes (N/P of 20:1) for different times (Blue: Hoechst 33 342 stained cell nuclei; Green: Lyso tracker green stained lysosome; Red: Cy3‐labeled pDNA).

With the unique properties, G3‐NGs 10:2 were employed as vectors to compact pDNA. By gel retardation assay, the compaction ability of G3‐NGs/pDNA at different N/P ratios (0.25:1 to 4:1) was determined (Figure 4a). G3‐NGs fully inhibited pDNA migration at the low N/P ratio of 0.5:1, suggesting that they possess excellent pDNA compaction capacity due to the unique structures. By the calculation, the maximum loading efficiency of pDNA in G3‐NGs is about 17.6%. Moreover, the hydrodynamic diameters and electrophoretic mobilities of G3‐NGs/pDNA polyplexes are critical parameters for efficient gene delivery. With the increase of N/P ratio (1:1 to 20:1), the size of G3‐NGs/pDNA polyplexes was almost constant, while their electrophoretic mobility decreased (Figures S15–S17). Several previous studies demonstrated that the polyplexes with a hydrodynamic diameter of around 200 nm and a positive surface potential of about 1.5 µmcm Vc^−1^ are amenable for gene delivery.^[^ ^38^, ^39^ ^]^

For gene delivery, the transfection efficiency and cytotoxicity of cationic dendrimers are strongly correlated with their generation.^[^ ^40^ ^]^ Generally, by virtue of high charge density, high‐generation dendrimers (e.g., G5) exhibit higher gene transfection and delivery efficacies, but also lead to an obviously increased toxicity on the transfected cells.^[^ ^20^ ^]^ Next, the biocompatibility and gene transfection of high‐generation G5 dendrimer and low‐generation G3‐NGs were compared. By CCK‐8 assay (Figure 4b), G5 dendrimer emerged obvious cytotoxicity for HeLa cells, less than 80% viability at the concentration of 50 µg mL^−1^. Although the viability of more than 80% was observed in G3‐NGs at the highest concentration of 500 µg mL^−1^. Notably, the cell viability in G3‐NGs/pDNA polyplexes (N/P = 20:1) was higher than that in G3‐NGs at the same concentration attributing to the reduction of positive charge after pDNA complexation.

Likewise, the delivery and expression efficiencies of G3‐NGs/pDNA polyplexes with different N/P ratios in HeLa cells were investigated using fluorescence microscopy (Figure 4d,e). The EGFP expression in cells (green fluorescence) increased with the N/P ratio of G3‐NGs/pDNA. Remarkably, at the N/P ratio of 20:1, the expression level of EGFP in HeLa cells after treatment with G3‐NGs/pDNA was much higher than that in HeLa cells after treatment with G5/pDNA. Of note, G5/pDNA displayed a higher transfection efficacy at the N/P ratio of 10:1 than that at the N/P ratio of 20:1. This is likely due to that severe cytotoxicity of high‐generation G5 dendrimer at high concentration impaired gene transfection efficiency. Next, the quantitative transfection efficiency of G3‐NGs/pDNA was calculated using flow cytometry to determine the percent of positive cells transfected with EGFP protein (Figures 4c, S18). Clearly, 0.1%, 2.2%, 12.5%, and 27.8% of HeLa cells were transfected by G3‐NGs/pDNA at N/P ratios from 1:1 to 20:1, respectively. At the N/P ratio of 20:1, 27.8% of cells after treatment with G3‐NGs/pDNA expressed EGFP, which is higher than 8.2% of cells after treatment with G5/pDNA. The observed partial cell rounding may result from cationic vector‐membrane interactions in the transfection process, that activates the RhoA/ROCK pathway and triggers cytoskeletal contraction (actin reorganization) and subsequent cell rounding. This represents a transient morphological change rather than cell death.^[^ ^41^, ^42^ ^]^ These results together suggested that low‐generation dendrimer NGs as gene vector possess an excellent transfection efficiency while ensuring low cytotoxicity when compared to high‐generation G5 dendrimer. Although the transfection efficiency of G3‐NGs/pDNA polyplexes is relatively low compared to other commercially available transfection agents, they have also gained some additional advantages due to their suitable size and unique performances, such as stimuli‐responsiveness, degradability, improved cell internalization, and tumor accumulation, as well as low cytotoxicity.

Furthermore, the intracellular trafficking and endosomal escape of G3‐NGs/pDNA polyplexes in HeLa cells were explored using confocal laser scanning microscope (CLSM) (Figure 4f). Cy3‐labeled pDNA was used in G3‐NGs/pDNA polyplexes (red fluorescence) which was cultured with cells for different times. With the incubation time increases, the amount of intracellular polyplexes increased. In the early stage of cell uptake, the localization of polyplexes and endo/lysosomes was overlapped. However, after 8 h, the polyplexes were separated from the endo/lysosomes, indicating that G3‐NGs/pDNA polyplexes display excellent endosomal escape ability, thus achieving satisfactory gene transfection efficiency.

In addition, we tried to evaluate the transfection efficiency of G3‐NGs/pDNA polyplexes in human umbilical vein endothelial cells (HUVEC), a cell line that is difficult to transfect. The result showed that G3‐NGs/pDNA polyplexes could also achieve DNA delivery in HUVEC, and 8.3% of HUVEC was transfected by G3‐NGs/pDNA (Figure S19). Although the transfection efficiency in HUVEC is lower than that in HeLa cells, it also indicates the potential of G3‐NGs as gene vector.

**Figure 5:**
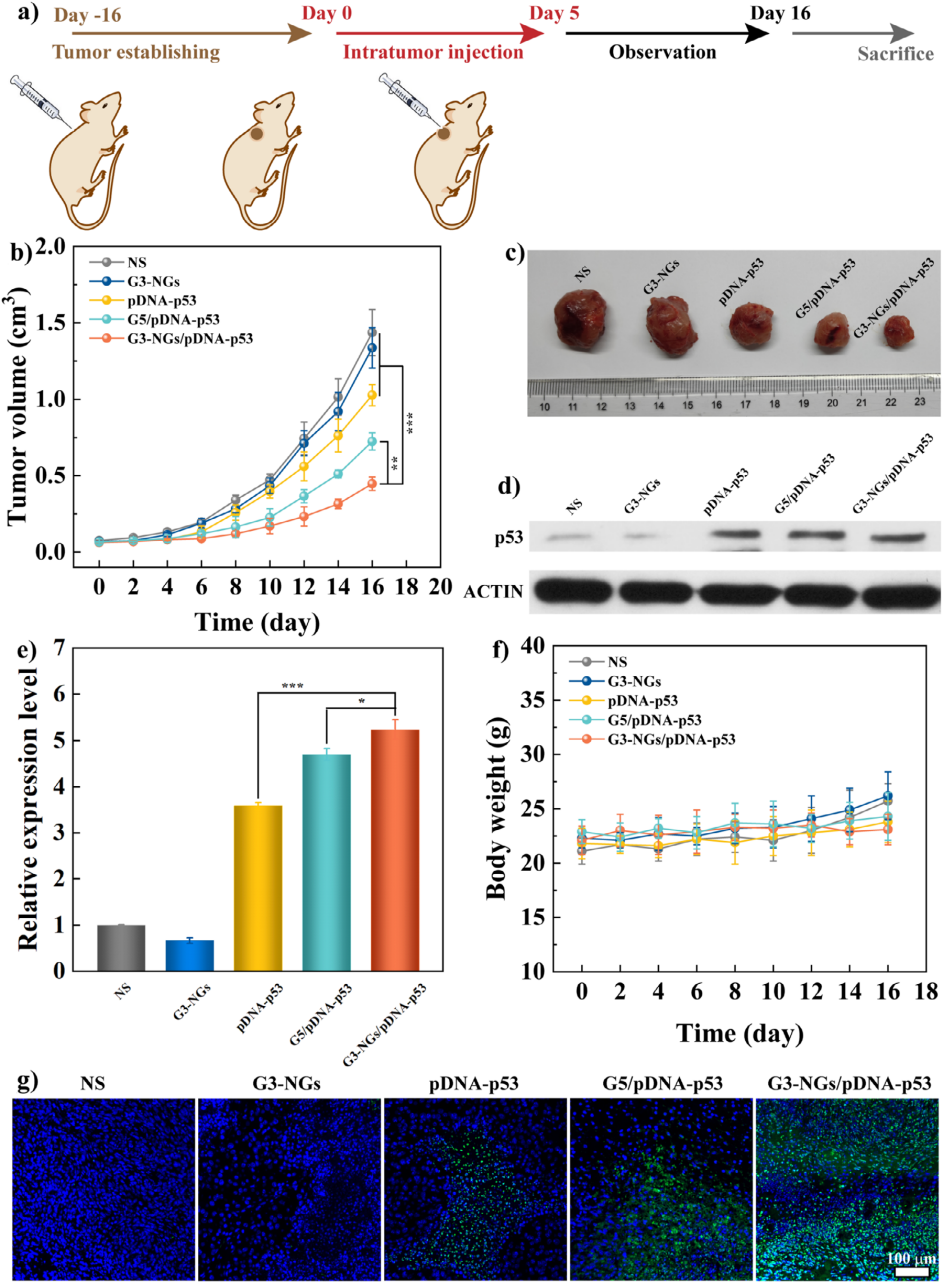
a) Schematic illustration of the administration process for gene therapy of tumor. b) Tumor volume change and c) tumor photo of mice bearing xenografted tumor in different groups for different periods (*n* = 5 mice in each group). d) Western blot assay of the expression level of proteins in xenografted tumor. The ACTIN protein is used as an internal control. Uncropped image of gel/blot is showed in Figure S20. e) Quantitative analysis of p53 protein expression level from the Western blot data. f) Body weight change of mice bearing xenografted tumor in different groups for different periods (*n* = 5 mice in each group). g) TUNEL staining of tumor sections in different groups. The normal tumor cells stained by DAPI (blue fluorescence) and the apoptotic tumor cells stained by TUNEL (green fluorescence).

Next, the tumor suppressor pDNA‐p53 was selected to prepare G3‐NGs/pDNA‐p53 polyplexes, which possesses an anticancer effect by inducing cell apoptosis, especially cell cycle arrest. This has been verified in our previous in vitro and in vivo experiments.^[^ ^39^ ^]^ The gene therapy efficacy of G3‐NGs/pDNA‐p53 polyplexes was assessed in animal model according to the treatment timeline (Figure 5a). Compared to the control groups, the tumor growth in both G3‐NGs/pDNA‐p53 and G5/pDNA‐p53 groups was greatly suppressed (Figure 5b,c). Among them, the G3‐NGs/pDNA‐p53 group showed the best tumor suppression effect. To verify the anti‐tumor mechanism, the p53 protein expression of tumor cells in different groups was measured by Western blot assay (Figures 5d,e, and S20). Apparently, the highest expression level of p53 proteins were observed in the tumor region of G3‐NGs/pDNA‐p53 group. These results suggested that the transfection of tumor suppressor pDNA‐p53 using G3‐NGs can induce the significant up‐regulation of p53 expression for tumor suppression. Meanwhile, the antitumor effect was further evaluated through TdT‐mediated dUTP Nick‐End Labeling (TUNEL) staining of tumor tissue in different groups (Figure 5g). The tumors after treatment of G3‐NGs/pDNA‐p53 polyplexes showed the largest necrosis and apoptosis areas (green fluorescence). Finally, the toxic side effects after treatment with G3‐NGs/pDNA‐p53 polyplexes was evaluated by tracking body weight change of mice and the hematoxylin and eosin (H&E) staining of the main organs. Similar to the control groups, the body weight of mice in G3‐NGs/pDNA‐p53 group had no obvious change (Figure 5f). Moreover, the negligible organ abnormality or damage in heart, liver, spleen, lung, and kidney was confirmed in G3‐NGs/pDNA‐p53 group (Figure S21). These results demonstrated that G3‐NGs/pDNA‐p53 polyplexes display highly efficient gene therapy and good biosafety in vivo simultaneously, which breaks up the transfection efficacy‐cytotoxicity correlation of cationic colloidal vectors in gene therapy. These positive outcomes benefit from the unique features of low‐generation dendrimer NGs (e.g., appropriate size, temperature‐responsiveness, polyampholyte property, and self‐triggered aminolysis) that can enhance EPR effect, protect gene from nuclease degradation, promote cellular internalization and lysosomal escape, as well as the controlled NG degradation along with pDNA release.

Next, we compared the developed G3‐NGs with other hyperbranched polymer‐based NGs. In the terms of gene compression, G3‐NGs can completely compress pDNA at a low N/P ratio of 0.5:1, while other NGs usually need to compress gene at the N/P ratio more than 1:1.^[^ ^43^, ^44^ ^]^ Among them, polyethylenimine‐based NGs obtained by the miniemulsion method can only compress siRNA even at an N/P ratio of 10:1,^[^ ^28^ ^]^ which is caused by the fact that the surfactant cannot be completely removed. Besides, the intracellular delivery and transfection of G3‐NGs/pDNA polyplexes are obtained at the N/P ratio of 5:1, which is a relatively value compared to other NGs.^[^ ^28^ ^]^ On the other hand, the NGs reported in the literature often show the increased toxicity with the increase of N/P ratio, leading to the reduction of intracellular delivery and transfection efficiency.^[^ ^45^, ^46^ ^]^ In contrast, G3‐NGs/pDNA polyplexes exhibit high transfection efficiency even at high N/P ratio due to their excellent biocompatibility. In addition, the hyperbranched polymer‐based NGs possess the concentration‐dependent cytotoxicity.^[^ ^47^ ^]^ Although researchers can improve NG biocompatibility by partial carboxylation or introducing biopolymers and liposomes,^[^ ^48^, ^49^, ^50^ ^]^ they still have certain cytotoxicity (cell viability <80%) at a relatively high concentration. Several degradable NGs are exploited to achieve higher biocompatibility, but at the expense of a small amount of transfection efficiency.^[^ ^44^ ^]^ In contrast, G3‐NGs/pDNA polyplexes exhibit negligible cytotoxicity (cell viability > 90%) at the concentration of 500 µg mL^−1^, and still retains good transfection efficiency. Moreover, compared with other NGs, we designed G3‐NGs exhibiting temperature‐responsiveness, polyampholyte property, and self‐triggered aminolysis. Importantly, with these excellent features, highly efficient gene therapy and good biosafety of G3‐NGs/pDNA polyplexes were systematically validated in vivo.

## Conclusion

In summary, for the first time, an emulsion‐free strategy based on covalently crosslinking of G3 dendrimer and PVCL‐GMA copolymer was developed to prepare G3‐NGs with high yield and purity, uniform size and morphology, and good colloidal stability, which cannot be achieved by the conventional miniemulsion method reported elsewhere.^[^ ^22^, ^51^ ^]^ Importantly, G3‐NGs display the unique characteristics of dual‐active groups, *o*‐hydroxyl amine unit, temperature‐responsiveness, polyampholyte property, and self‐triggered aminolysis. Therefore, G3‐NGs can “temporarily” acquire high charge density through the covalent crosslinking of low‐generation dendrimer for improving DNA compression, protecting DNA from nuclease degradation, promoting cell internalization and lysosomal escape, as well as efficient DNA delivery, followed by self‐triggered aminolysis into small dendrimers to control DNA release, reduce cytotoxicity, and facilitate metabolism in vivo. As a proof‐of‐concept study, G3‐NGs address the dilemma between transfection efficacy and cytotoxicity of cationic colloidal vectors in gene therapy. Compared to high‐generation dendrimers, G3‐NGs have also several unique advantages, such as lower cost, higher gene transfection efficacy, and better biosafety. We therefore believe that the developed strategy will provide innovative thought for the design of the next generation of smart gene vectors for clinical translation.

## Conflict of Interests

The authors declare no conflict of interest.

## Supporting information



Supporting information

## Data Availability

The data and code that support the findings of this study are available from the corresponding author upon reasonable request.
