# Supplementary material for: Functional Dendrimer Nanogels for DNA Delivery and Gene Therapy of Tumors
Source: Angew Chem Int Ed Engl. 2025 May 5;64(30):e202505669. doi: 10.1002/anie.202505669 (PMC12281078; doi:10.1002/anie.202505669)
Supplement: Supplementary file 1 — Supporting information [file ANIE-64-e202505669-s001.docx]

Supporting Information

Functional Dendrimer Nanogels for DNA Delivery and Gene Therapy of Tumors

Xin Li,* Zhijun Ouyang, Laura Hetjens, Ming Ni, Kuailu Lin, Yong Hu, Xiangyang Shi,* and Andrij Pich*

DOI: 10.1002/anie.2016XXXXX

Experimental Procedures

**Materials**

*N*-Vinylcaprolactam (VCL), glycidyl methacrylate (GMA), anisole, ethanol, dimethylsulfoxide (DMSO), diethyl ether, potassium ethyl xanthogenate, dichloromethane, and 2,2’-Azobis(2-methylpropionitrile) (AIBN) were obtained from Sigma-Aldrich. Methyl 2-bromopropionate supplied by Acros Organics. Ethylenediamine core G3.NH_2_ and G5.NH_2_ PAMAM dendrimers were purchased from Weihai CY Dendrimer Technology Co., Ltd.. Primary Amino Nitrogen (PANOPA) Assay Kit was supplied from Megazyme. Enhanced green fluorescent protein plasmid DNA (pDNA-EGFP) and tumor suppressor p53 plasmid DNA (pDNA-p53) were provided by Shanghai Gene Pharma. Cell Counting Kit-8 (CCK-8) was from Beyotime Institute of Biotechnology.

**Synthesis of PVCL-GMA and G3-PVCL-GMA copolymers**

First, Methyl 2-(ethoxycarbonothioylthio) propanoate was synthesized as the chain transfer agents (CTA) according to our previous work.^[1]^ Then, the PVCL-GMA copolymer was prepared using the RAFT polymerization method. The VCL (2 g, 14.37 mmol) and CTA (0.042 g, 0.21 mmol) dissolved in 5 mL of anisole was placed into a 25 mL Schlenk flask. The solution was degassed by five freeze-pump-thaw cycles. After that, the GMA (0.23 g, 1.6 mmol) dissolved in 1 mL of anisole and AIBN (0.0058 g, 0.035 mmol) dissolved in 0.2 mL of anisole, respectively. The solutions were purged with dry nitrogen for 1 h. The AIBN solution was added to the Schlenk flask immediately, while the GMA solution was supplemented continuously with a syringe pump (Harvard Apparatus, Holliston, MA) in 3-5 h to ensure the homogeneous distribution of GMA in copolymer. Afterward, the polymerization was immediately stopped by immersing in liquid nitrogen. The PVCL-GMA copolymer was precipitated in an excess amount of cold diethyl ether, further washed with diethyl ether, dried under vacuum at 40 °C for 48 h. The yield of PVCL-GMA copolymers is about 67.8%.

Next, G3 PAMAM dendrimer (50 mg, 0.007 mmol) was dispersed in DMF (5 mL), and then the PVCL-GMA copolymer (150 mg, 0.028 mmol) dissolved in DMF (2 mL) was dropwise added under vigorous stirring at the temperature of 60^o^C for 8 h. The mixture was dialyzed (MWCO = 12000-14000) in DMF for 3 days. The solvent was removed under vacuum, and the G3-PVCL-GMA copolymer was obtained by the lyophilization. The yield of G3-PVCL-GMA copolymer is about 88.3%.

**Synthesis of G3-NGs**

G3 PAMAM dendrimer with different contents were employed as the crosslinker to synthesize the G3 crosslinked NGs by the emulsion-free method. The G3-PVCL-GMA copolymer (20 mg) was reacted with G3 dendrimer at different mass ratios of 10:1, 10:2, and 10:5 in 2 mL base buffer (NaCO_3_-NaHCO_3_ buffer, pH 9-10), and then stirred for 24 h. After that, the mixtures were centrifuged by ultrafiltration with the MWCO of 5000 (5000 rpm, 5 min), and then freeze-dried to obtain the G3 crosslinked NGs, named as G3-NGs 10:1, G3-NGs 10:2, and G3-NGs 10:5, respectively. For subsequent experiments, the solution of NGs is freshly prepared.

**Characterization techniques**

Gel permeation chromatography (GPC) was performed on a combined GPC system with a high-performance liquid chromatography pump, a Jasco 2035-plus RI detector and four MZ-DVB gel columns. The solvent used is DMF. The molecular weight was calculated using a polystyrene calibration. FTIR spectra was collected on a Nicolet NEXUS 670 Fourier Transform IR spectrometer. ^1^H NMR spectra were carried out on a Bruker DPX-400 FT NMR spectrometer at 400 MHz. The solvent used is CDCl_3_. Dynamic light scattering (DLS) measurement was conducted using a Malvern Zetasizer Nano. Scanning transmission electron microscopy (STEM) image was performed on ultra-high-resolution scanning electron microscope SU 9000 operating at a voltage of 30 kV.

**Temperature-responsive property**

Could points of G3-NGs were determined by turbidity measurement in a CARY 100 Bio UV-visible spectrophotometer. These samples (0.5 mg/mL) were scanned with a temperature trend from 5^o^C-70^o^C and with a fixed wavelength of 400 nm. Moreover, the G3-NGs were dissolved in water, and their hydrodynamic diameter and electrophoretic mobility depending on the temperatures were performed on Malvern Zetasizer Nano ZS at the temperature of 5^o^C-60^o^C respectively. The values of hydrodynamic diameter and electrophoretic mobility were obtained using ZS XPLORER software.

**Polyampholyte property**

The electrophoretic mobilities of G3-NGs were measured on Malvern Zetasizer Nano ZS at 25 ^o^C as a function of pH from 3-11. Before measurement, the aqueous dispersion of samples was diluted with PBS buffers to adjust pH from 3 to 11. The values of electrophoretic mobility were obtained using ZS XPLORER software.

**Self-triggered degradation**

The degradation of G3-NGs by self-triggered aminolysis was conducted by monitoring the change of hydrodynamic diameter and STEM image. The G3-NG was dispersed in pH 6.8 buffer (2 mg/mL) and then stirred at 37^o^C for different times, and the hydrodynamic diameter during NG degradation was measured as a function of time by DLS measurement. Likewise, the NG morphology before and after degradation was characterized using STME image.

**pDNA compression within NGs (G3-NGs/pDNA polyplexes)**

The pDNA (pDNA-EGFP or pDNA-p53) was incubated with appropriate amount of G3-NGs 10:2 at different N/P ratios from 0.25:1 to 4:1 in PBS for 30 min to form the G3-NGs/pDNA polyplexes. Furthermore, the gel retardation assay of NGs/pDNA polyplexes with different N/P ratios was characterized. The gel was prepared by dissolving 1% (w/v) agarose gel and 4S Green Plus Nucleic Acid Stain in Tris-acetate-EDTA buffer, and melted under a microwave. The polyplexes (20 μL) were added into the wells of the gel, and electrophoresis was performed at 85 V for 30 min. The retardation of pDNA was visualized using a UV transilluminator. Likewise, the hydrodynamic size and electrophoretic mobility of G3-NGs/pDNA polyplexes with different N/P ratios were measured.

**Cytotoxicity assay**

Cell Counting Kit-8 (CCK-8) assay was used to evaluate the cytotoxicity of G3-NGs, G5, and G3-NGs/pDNA polyplexes at different concentrations. HeLa cells were seeded into a 96-well plate at a density of 1.0 × 10^4^ cells/well with DMEM medium the day before the experiment. Then the medium in each well was replaced with 0.1 mL fresh medium containing PBS or different samples (G3-NGs, G5, or NGs/pDNA polyplexes, 10 μL) with the final concentration ranging (10-500 μg/mL). The cells were incubated at 37 ^o^C and 5% CO_2_ for another 24 h. Then the cells were washed 3 times with PBS, and the CCK-8 (10 μL) was added into each well with serum-free and antibiotics-free medium (100 μL) and then the cells were incubated continuously for another 3 h. After that, the absorbance at 450 nm in each well was recorded using a Multiskan MK3 ELISA reader. The statistical data are presented as average values ± s.d. (n = 3 independent experiments).

**pDNA transfection efficacy in vitro**

HeLa cells were seeded in 24-well plates at a density of 1.0 × 10^5^ cells/well with DMEM medium the day before the experiment. The medium in each well was then replaced with serum-free and antibiotics-free DMEM (0.5 mL) containing PBS (0.2 mL), pDNA, G3-NGs/pDNA or G5/pDNA polyplexes with 1 μg pDNA at different N/P ratios from 1:1 to 20:1. The cells were furthermore incubated for another 4 h. Afterwards, the cells were washed 3 times, and the serum-free and antibiotics-free medium was added for continuously incubating for another 24 h.

In addition, human umbilical vein endothelial cells (HUVEC) were seeded in 24-well plates at a density of 1.0 × 10^5^ cells/well with DMEM medium the day before the experiment. The medium in each well was then replaced with serum-free and antibiotics-free DMEM (0.5 mL) containing G5/pDNA polyplexes with 1 μg pDNA at N/P ratios of 20:1. The cells were furthermore incubated for another 4 h. Afterwards, the cells were washed 3 times, and the serum-free and antibiotics-free medium was added for continuously incubating for another 24 h.

For pDNA transfection, the EGFP expression of the transfected cells was observed by an Axio Vert.Al Carl Zeiss fluorescence microscope (Ex: 488 nm). Moreover, the quantitative analysis of pDNA transfection in cells was investigated using flow cytometry with a Becton Dickinson Facscan analyzer. The statistical data are presented as average values ± s.d. (n = 3 independent experiments).

**Intracellular trafficking and lysosomal escape in vitro**

Cy3-labeled pDNA was used for intracellular trafficking and localization of G3-NGs/pDNA polyplexes. 2×10^5^ HeLa cells/well were seeded in confocal dish cultured overnight. Then the medium in confocal dish was replaced with serum-free and antibiotics-free DMEM (1 mL) containing G5/pDNA polyplexes with 2 μg pDNA at N/P ratios of 20:1. The cells were furthermore incubated for another 4 h. Then cells were cultured with fresh complete medium. After 2, 4, 6 or 8 h, the cells in confocal dish were co-incubated with different dyes, respectively. Incubation with Lyso tracker green for 20 min was used to label lysosomes, and incubation with Hoechst 33342 for 7 min was used to stain the cell nuclei. Finally, the cells were washed with PBS for 3 times and samples were scanned using a 63× oil immersion objective lens by using a ZEISS LSM-700 laser scanning confocal microscope.

**Gene therapy and systemic toxicity in vivo**

All animal experiments were approved by the Ethical Committee for Animal Care of Tongji University and also followed the policy of the National Ministry of Health (TJAF00124101). The HeLa xenograft tumor model with a volume of 0.05-0.07 cm^3^ was established on each BALB/c nude mouse. The xenograft tumor-bearing mice were randomly divided into 5 groups with five mice in each group: Saline (NS), G3-NGs, pDNA-p53, G3-NGs/pDNA-p53 and G5/pDNA-p53 polyplexes. On day 0-5, the mice were intratumorally injected with NS, G3-NGs, pDNA-p53, G3-NGs/pDNA-p53 or G5/pDNA-p53 polyplexes (pDNA = 40 or 20 μg), and then the mice were sacrificed on day 6 and tumor tissues were processed for Western blot analysis according to the previous work.^[2]^ Moreover, the tumor tissues also were processed for TdT-mediated dUTP Nick-End Labeling (TUNEL) staining according to the previous work.^[3]^

The major organs (heart, liver, spleen, lung, and kidney) in each group also were harvested on day 6, fixed in 4% paraformaldehyde, embedded in paraffin and then sectioned into slices for hematoxylin and eosin (H&E) staining according to the previous work.^[4]^

Throughout the observation process, the relative tumor volume and body weight of all mice were recorded at the required time point. The tumor volume (*V*) calculated by the Eqs. of $V=W^{2}\times L/2$. Where *V* are the tumor volume after treatment, *W* and *L* are the width and length of the tumor, respectively.

**Statistical analysis**

One-way analysis of variance statistical analysis was performed to evaluate the significance of the experimental data. A p value of 0.05 was selected as the significance level, and the data were indicated with (*) for p < 0.05, (**) for p < 0.01, and (***) for p < 0.001, respectively.

Results and Discussion

**Table S1.** GPC analysis of G3 dendrimer, PVCL-GMA and G3-PVCL-GMA copolymers.

| **Sample** | **Mn**  **(g/mol)** | **Mw**  **(g/mol)** | **Đ** |
| --- | --- | --- | --- |
| G3 | 3279 | 4531 | 1.12 |
| PVCL-GMA | 5309 | 7378 | 1.27 |
| G3-PVCL-GMA | 19982 | 23146 | 1.12 |


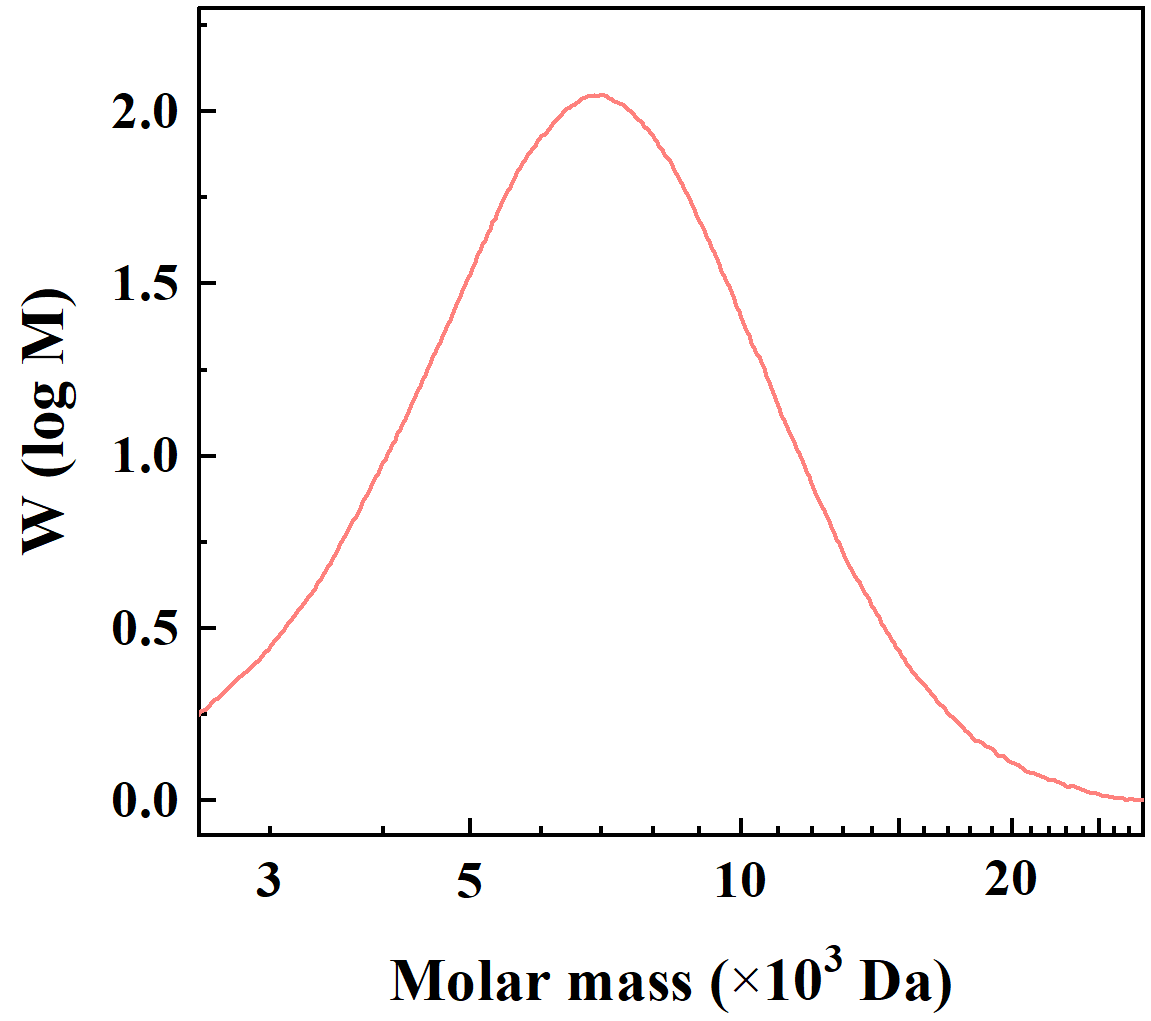


**Figure S1.** GPC measurement of PVCL-GMA copolymer.





**Figure S2.** FTIR spectra of PVCL-GMA copolymer. The peaks at 1630 cm^-1^ and 1725 cm^-1^ belong to the C=O from VCL and GMA part, respectively.


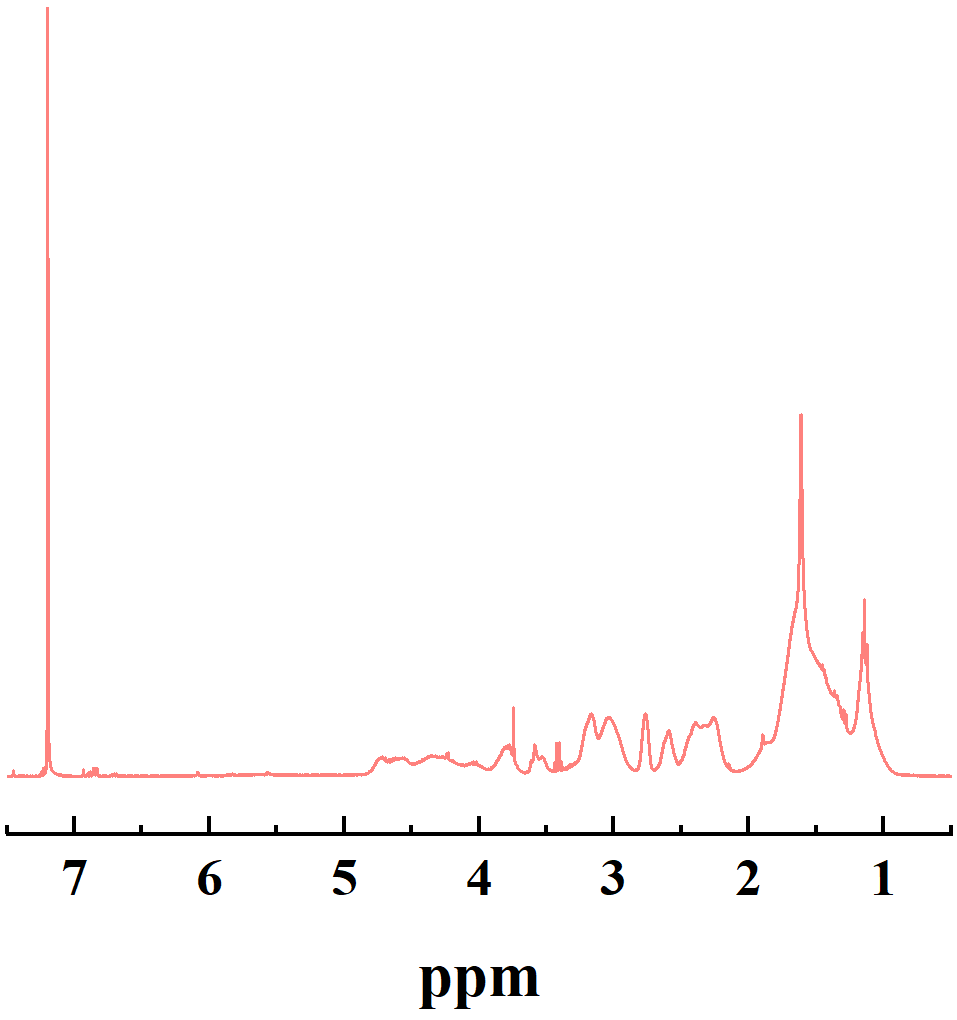


**Figure S3.** ^1^H NMR spectra of PVCL-GMA copolymer. The characteristic signals at (2.4 ppm, 3.2 ppm, and 4.3 ppm) and at (2.8 ppm, 3.1 ppm, and 3.8 ppm) are assigned to the VCL and GMA part, respectively.


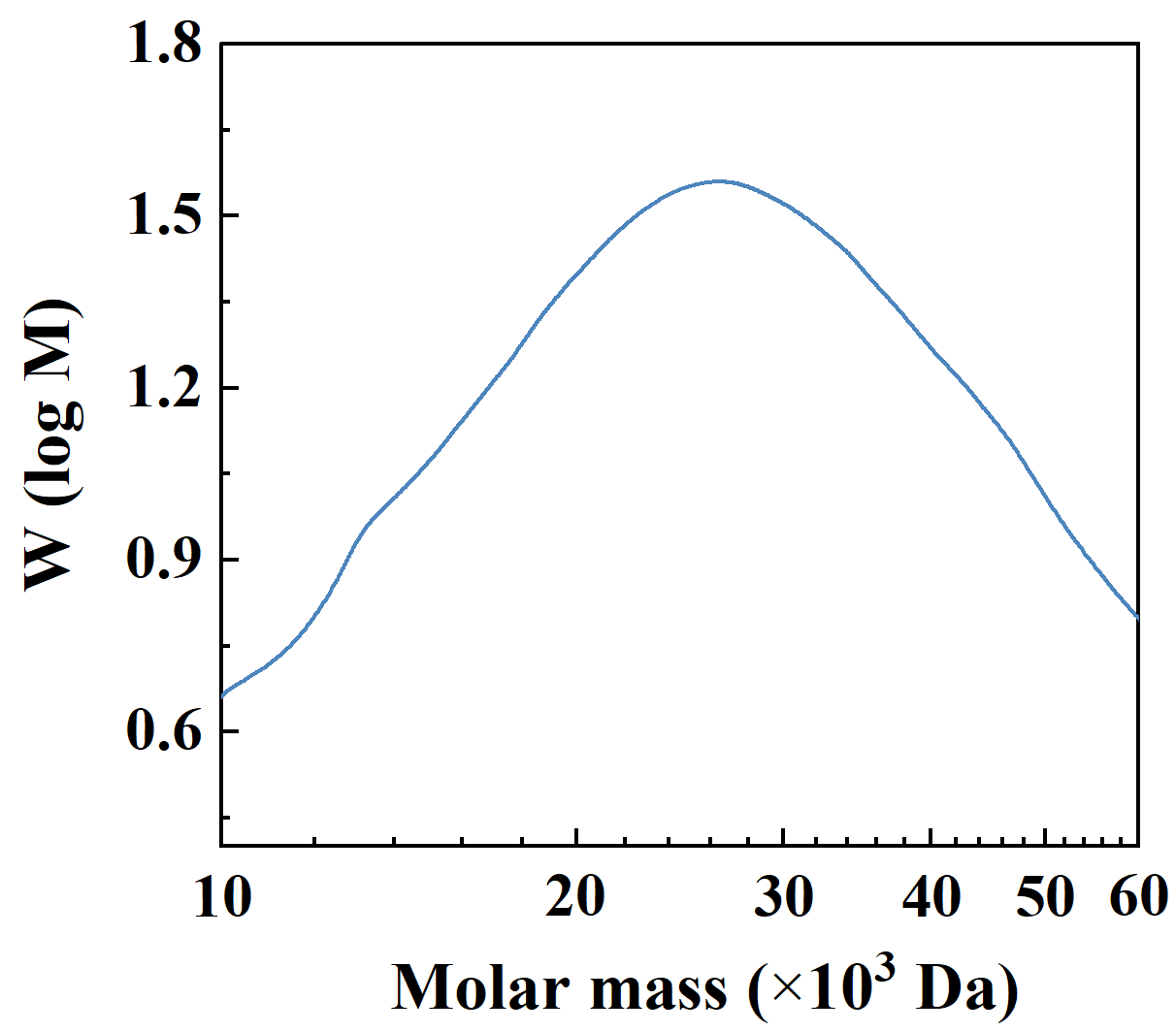


**Figure S4.** GPC measurement of G3-PVCL-GMA copolymer.


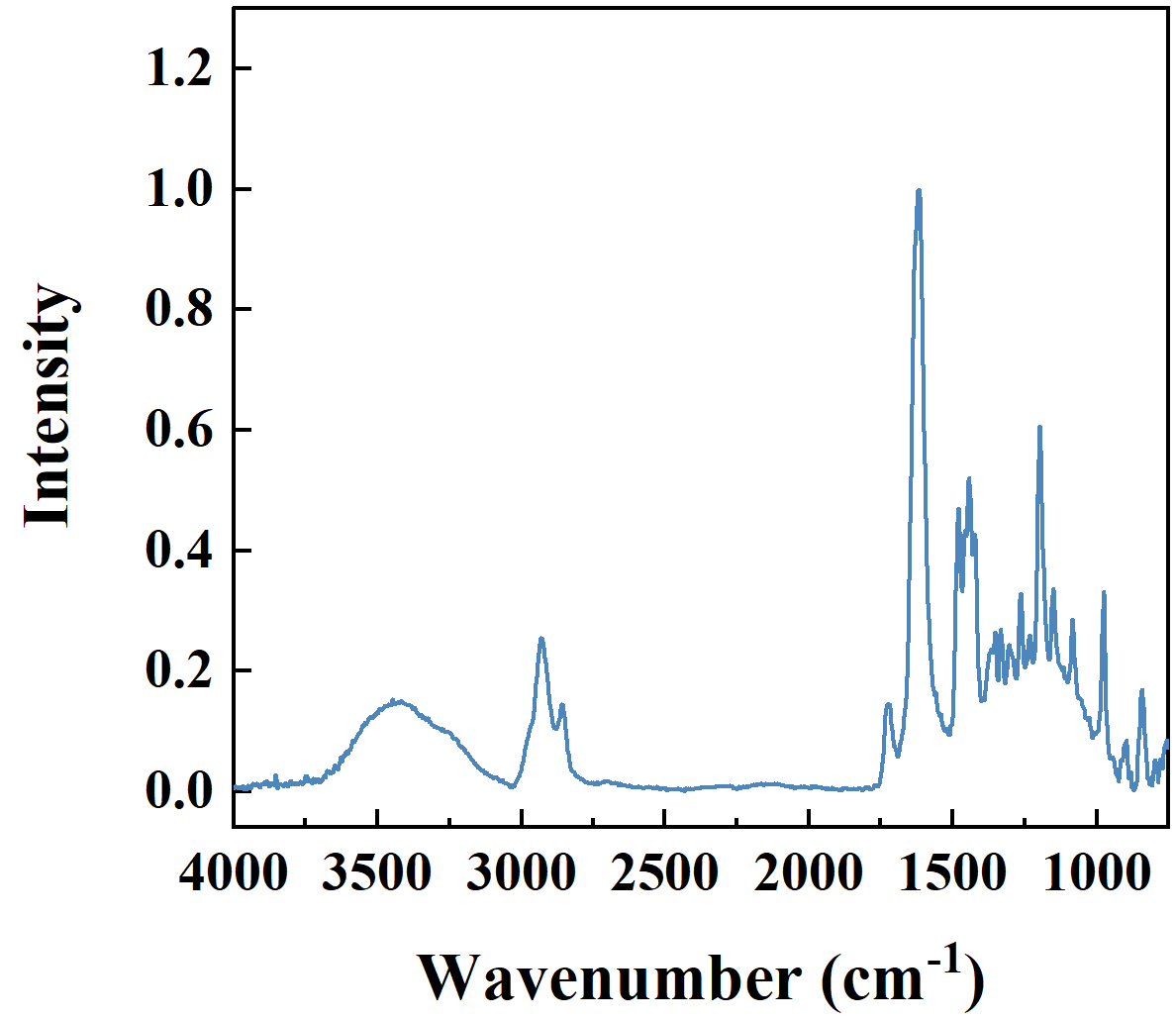


**Figure S5.** FTIR spectra of G3-PVCL-GMA copolymer. Compared to PVCL-GMA copolymer, the peak intensities of vGMA (1725 cm^-1^) and vN-H (1562 cm^-1^) from G3-PVCL-GMA copolymer are weakened and enhanced, respectively.


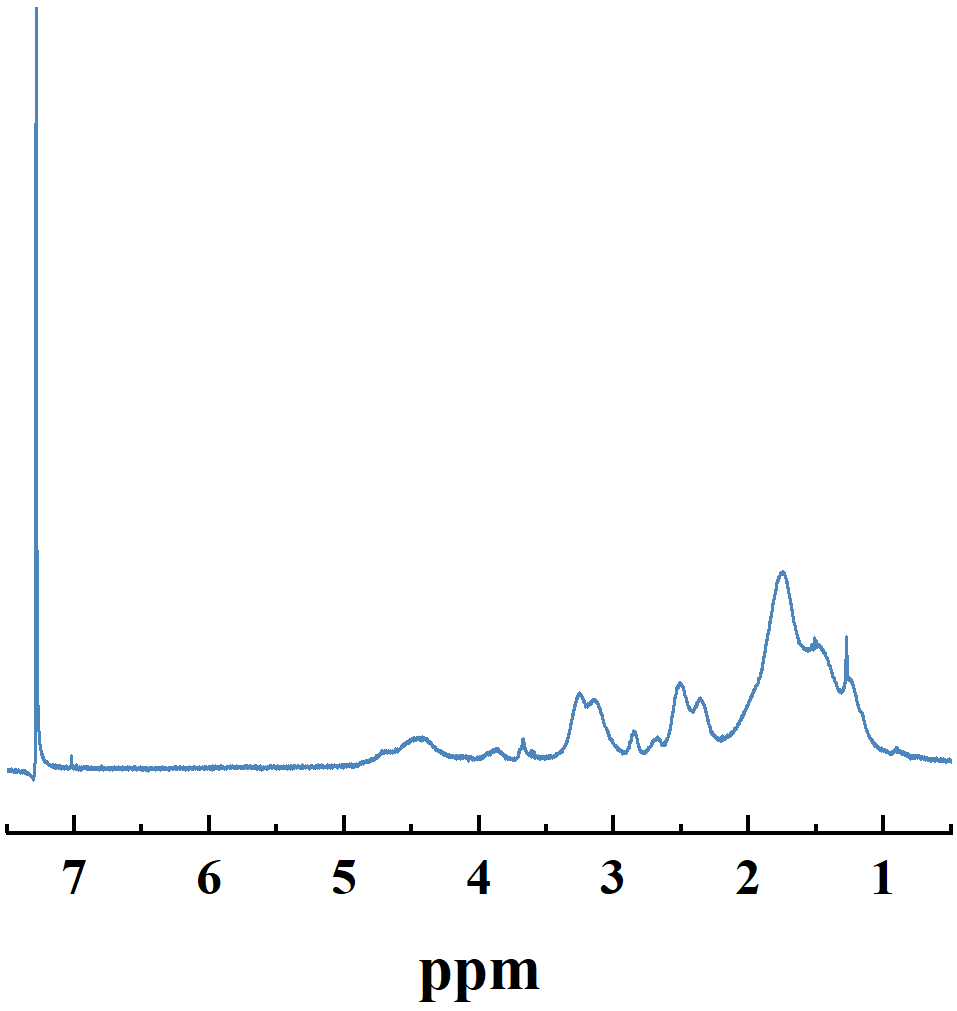


**Figure S6.** ^1^H NMR spectra of G3-PVCL-GMA copolymer. Compared to PVCL-GMA copolymer, the signals of G3-PVCL-GMA copolymer at 2.8 ppm from GMA are intensified due to the formation of *o*-hydroxyl amine unit.





**Figure S7.** Chemical structure of G3 PAMAM dendrimer.


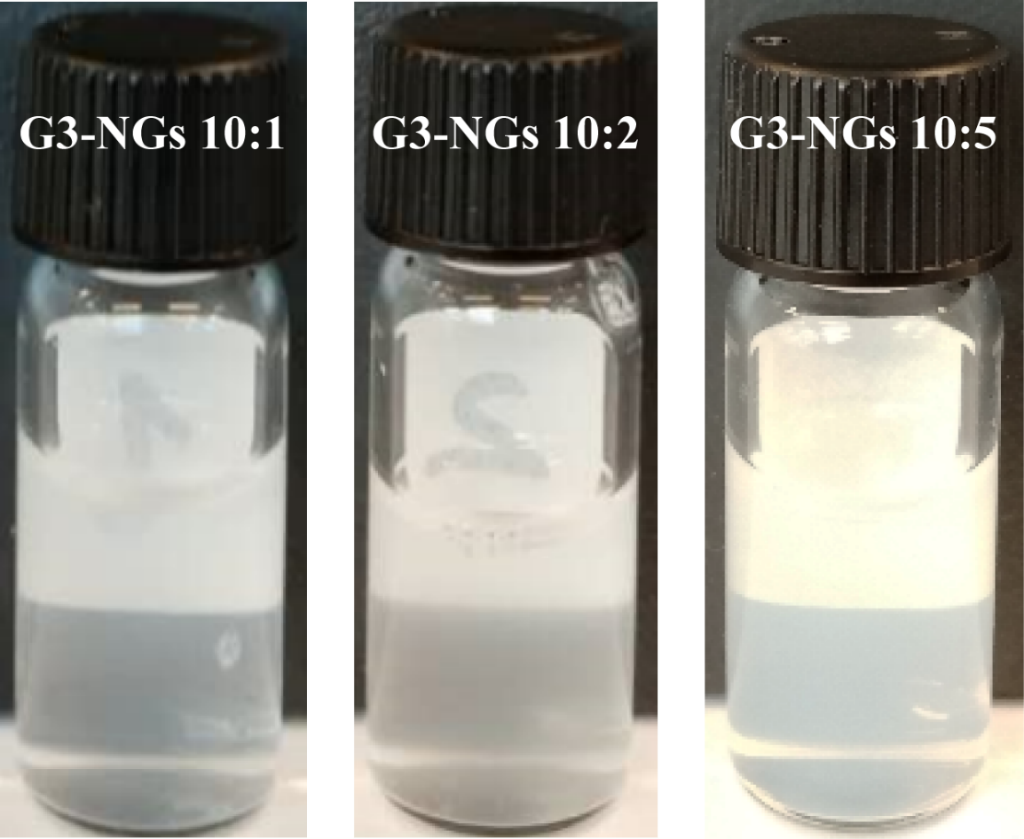


**Figure S8.** Digital photos of the aqueous solution of G3-NGs.





**Figure S9.** Hydrodynamic diameter profiles with number distribution of G3-NGs.


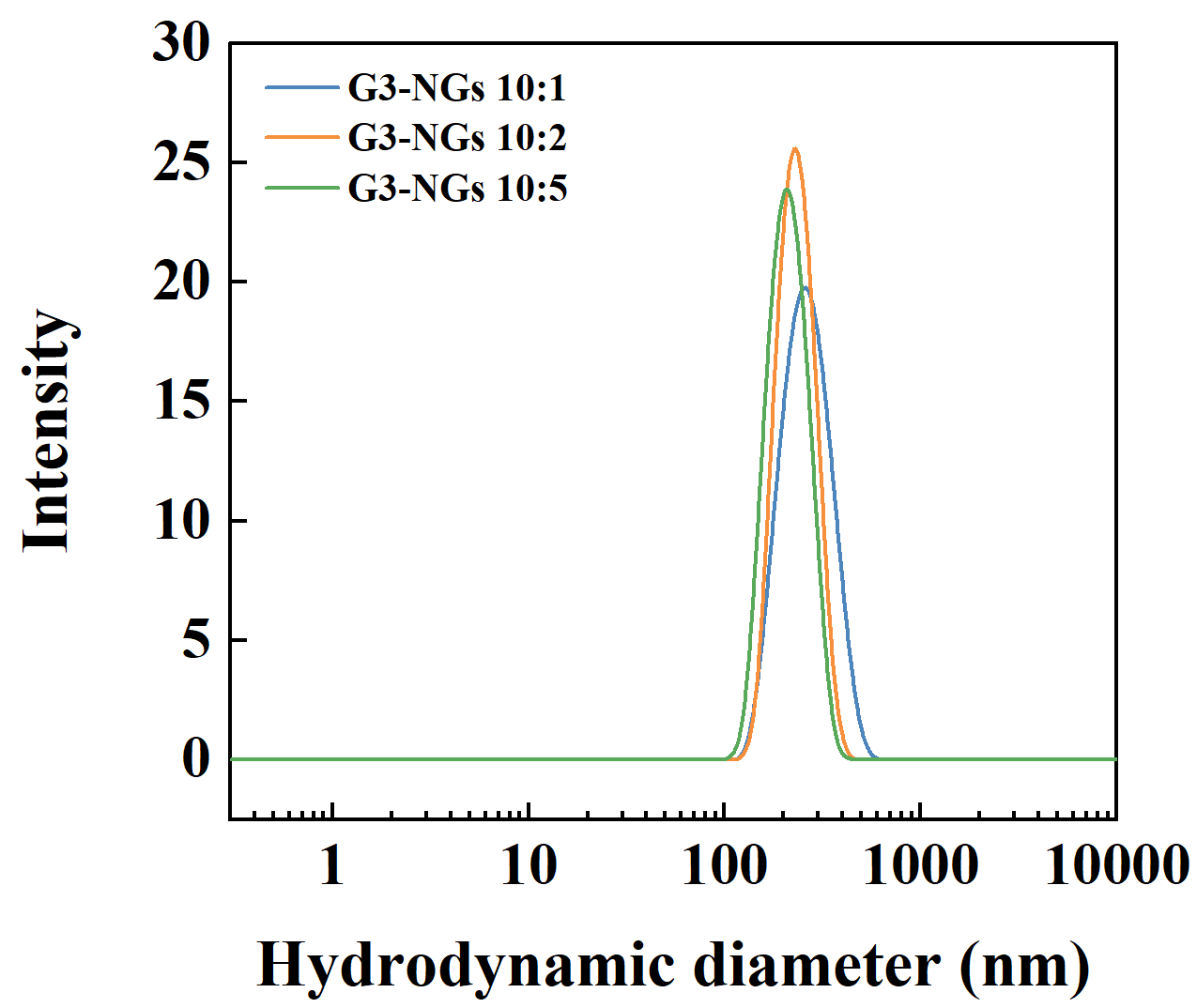


**Figure S10.** Hydrodynamic diameter profiles with intensity distribution of G3-NGs.


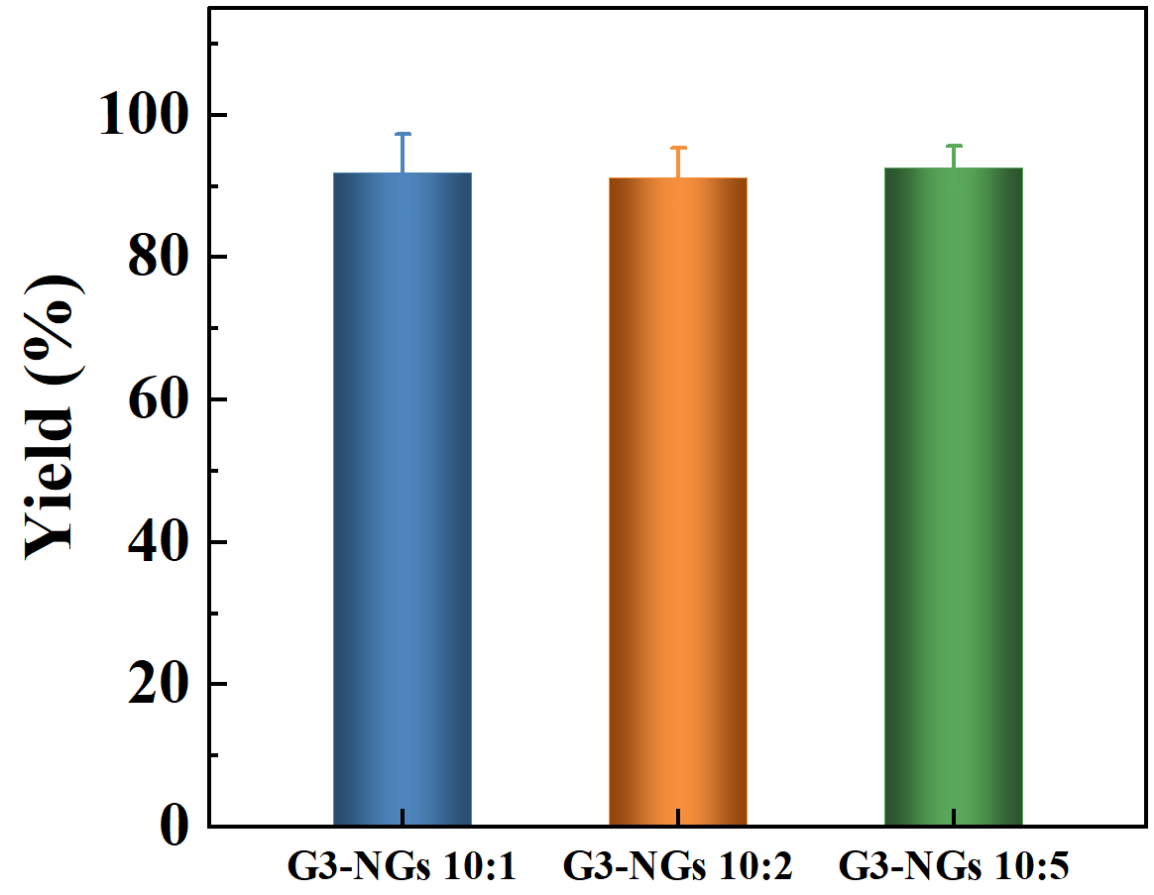


**Figure S11.** The yields of the synthesized G3-NGs.





**Figure S12.** The element contents of C, N, O in G3-NGs from elemental mapping images.





**Figure S13.** Hydrodynamic diameters of G3-NGs at different temperatures.





**Figure S14.** Electrophoretic mobilities of G3-NGs at the temperatures from 5-60 ^o^C.


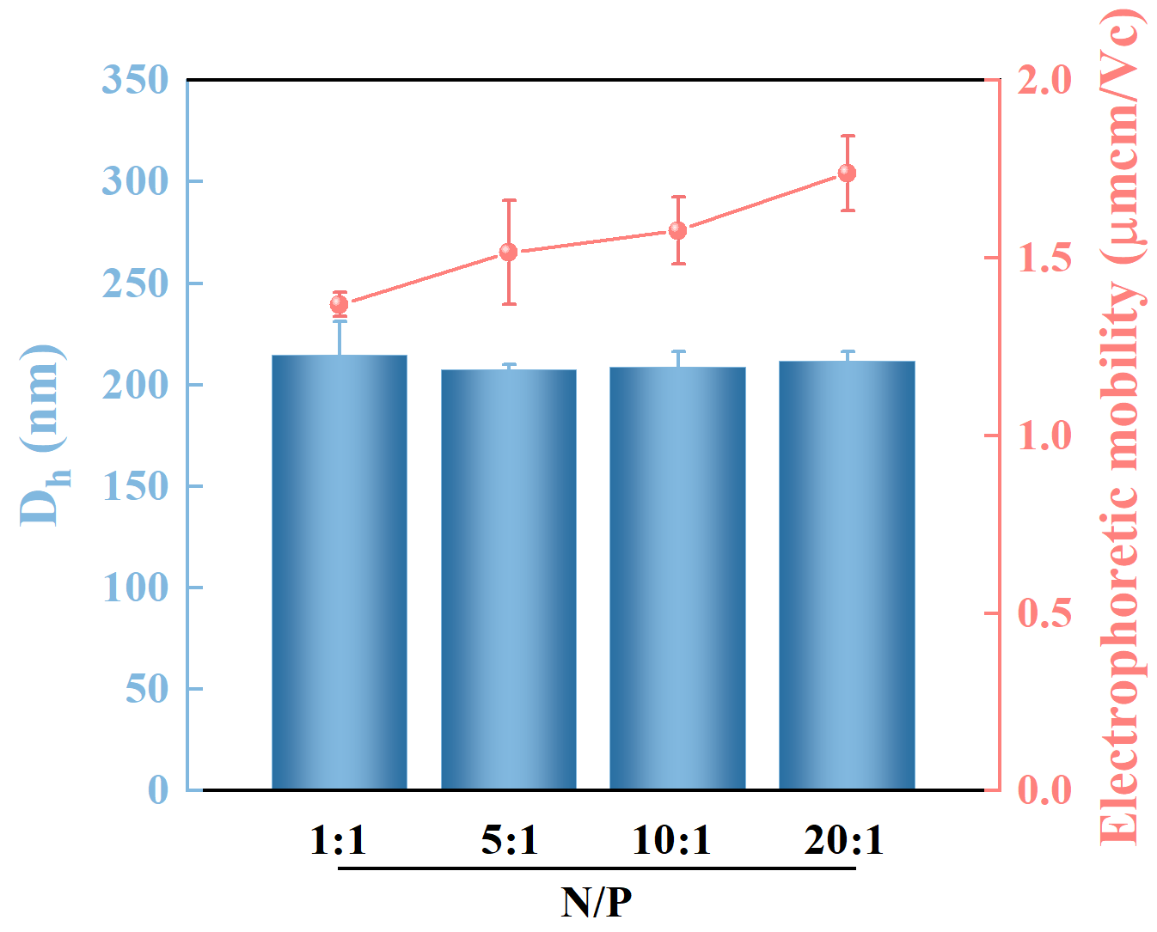


**Figure S15**. Hydrodynamic diameters and electrophoretic mobilities of G3-NGs/pDNA polyplexes at different N/P ratios.


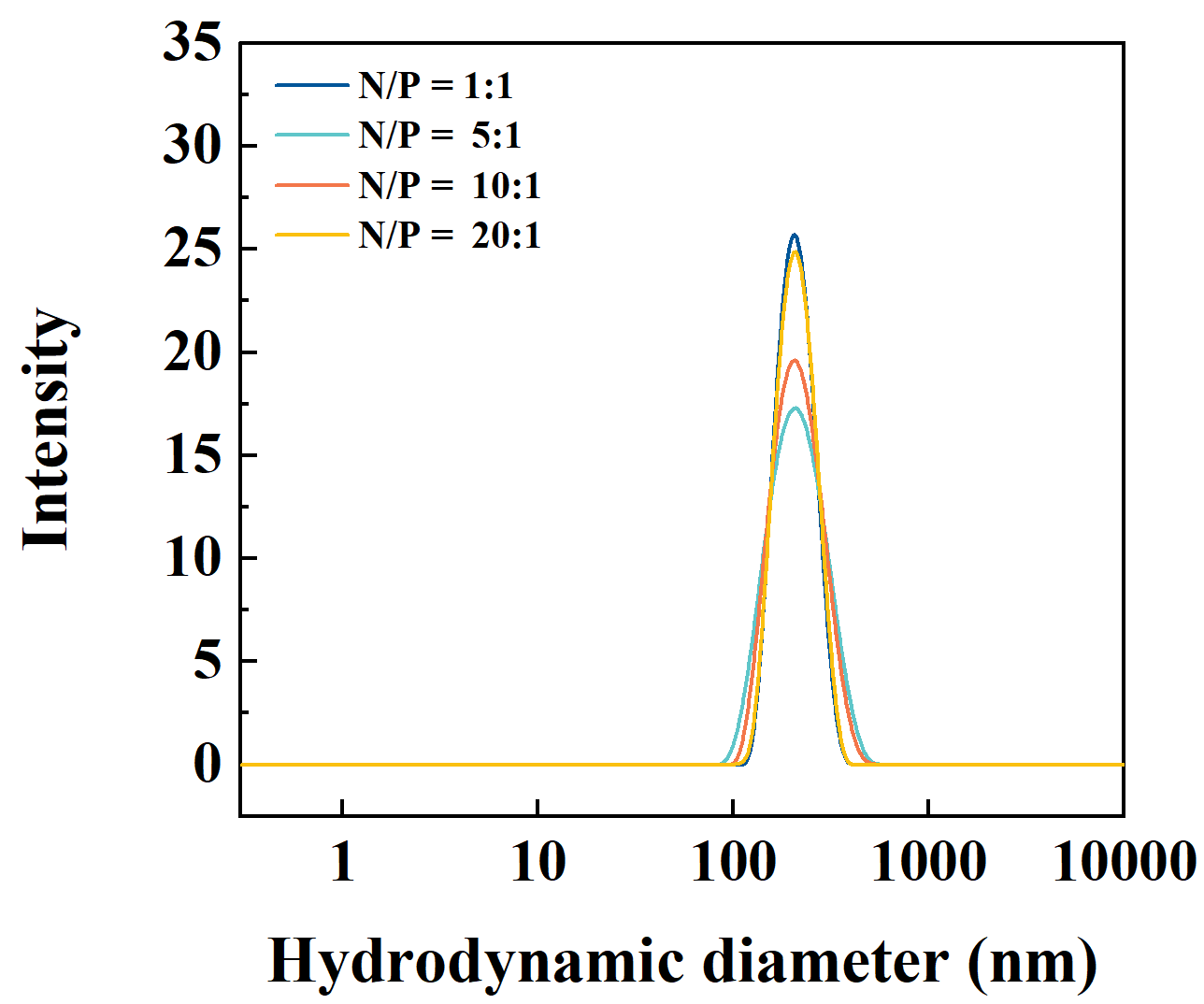


**Figure S16.** Hydrodynamic diameter profiles with intensity distribution of G3-NGs/pDNA polyplexes at different N/P ratios.


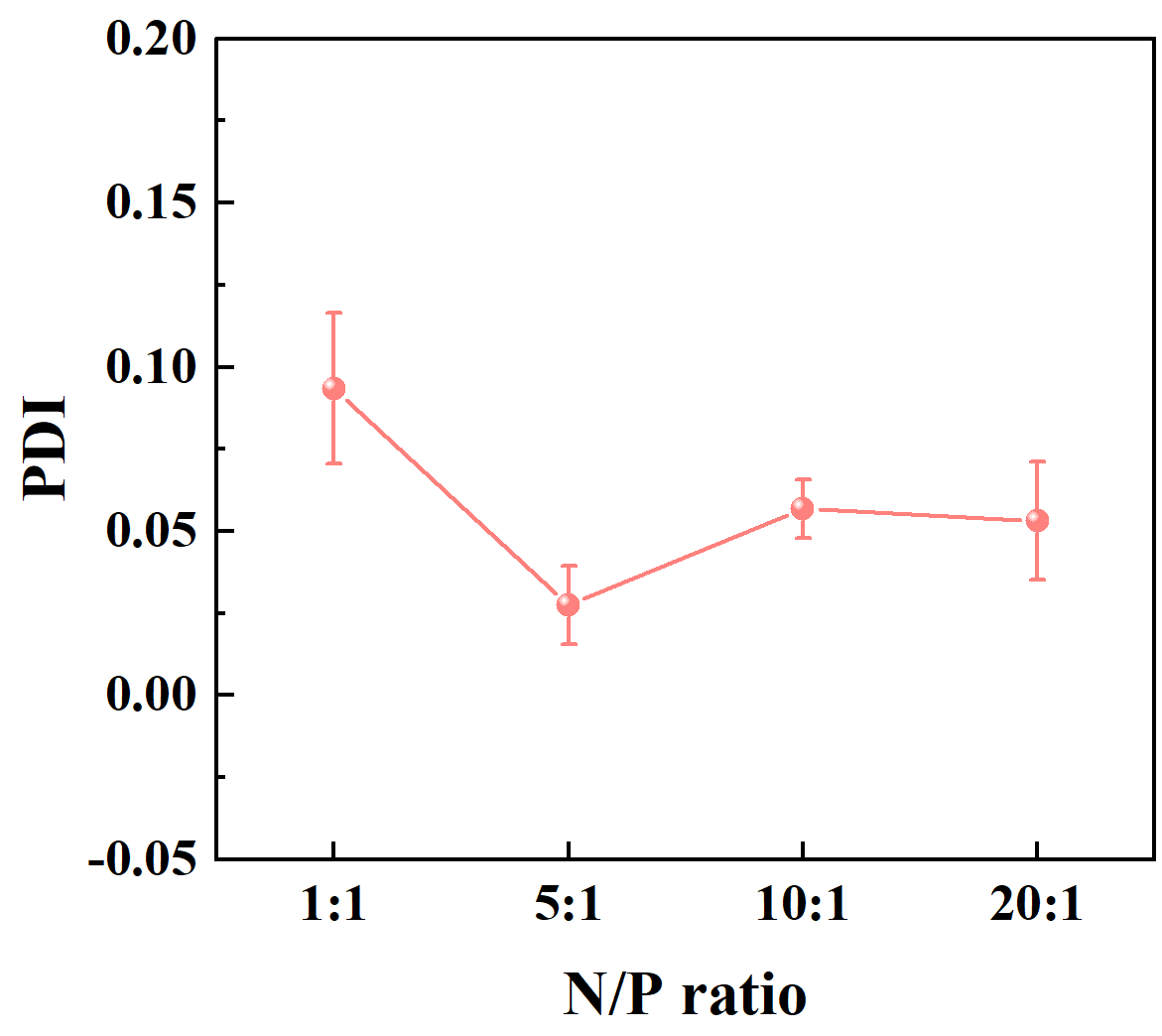


**Figure S17.** PDIs of G3-NGs/pDNA polyplexes at different N/P ratios.


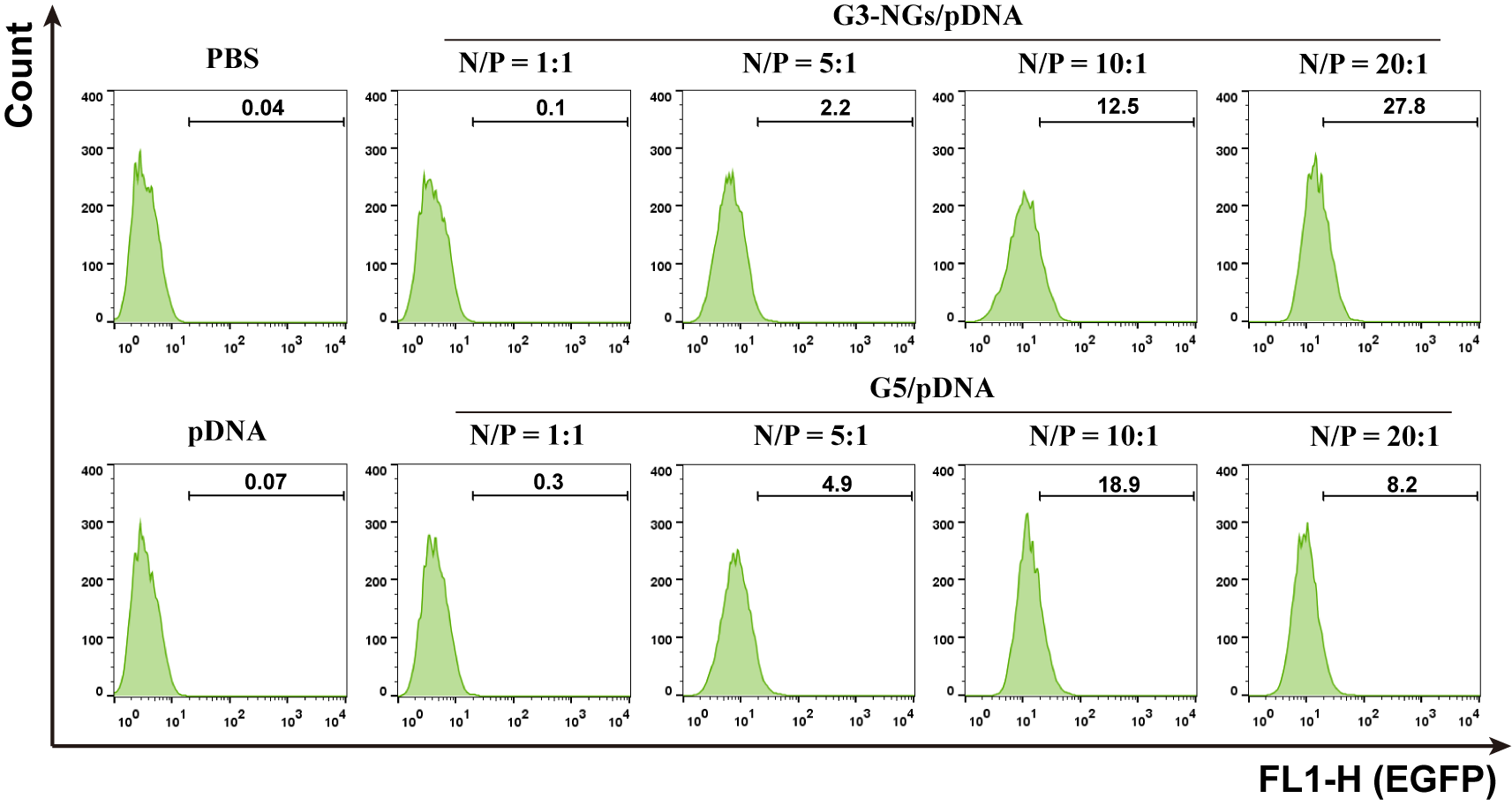


**Figure S18.** Flow cytometry analysis of the ratio of transfected cells.


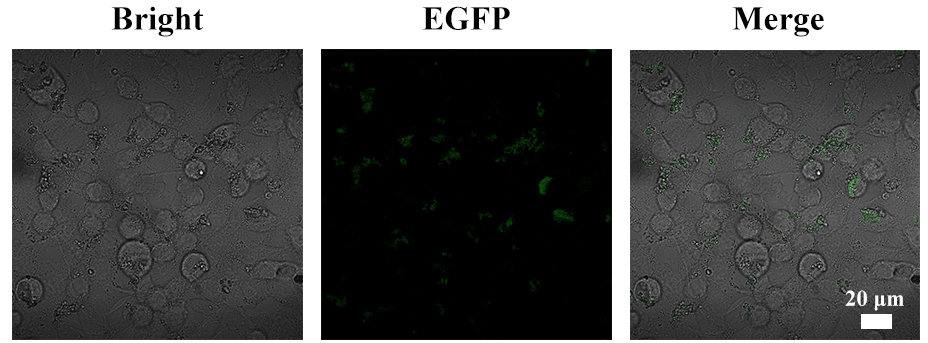


**Figure S19.** Fluorescent microscopy image of EGFP transfection in HUVEC with G3-NGs/pDNA polyplexes at an N/P ratio of 20:1.


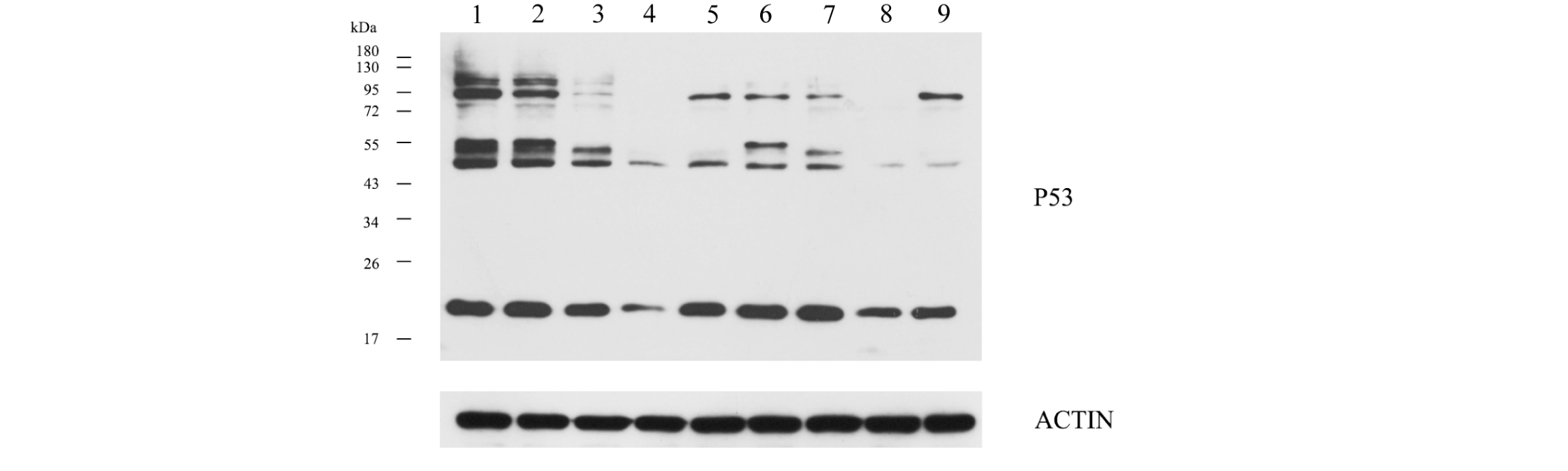


**Figure S20.** Uncropped image of gel/blot for Figure 5d. Among them, 1-4 represent G3-NGs/pDNA-p53, G5/pDNA-p53, pDNA-p53, and G3-NGs groups respectively (pDNA = 40 μg); 5-8 represent G3-NGs/pDNA-p53, G5/pDNA-p53, pDNA-p53, and G3-NGs groups respectively (pDNA = 20 μg); 9 represents NS group.


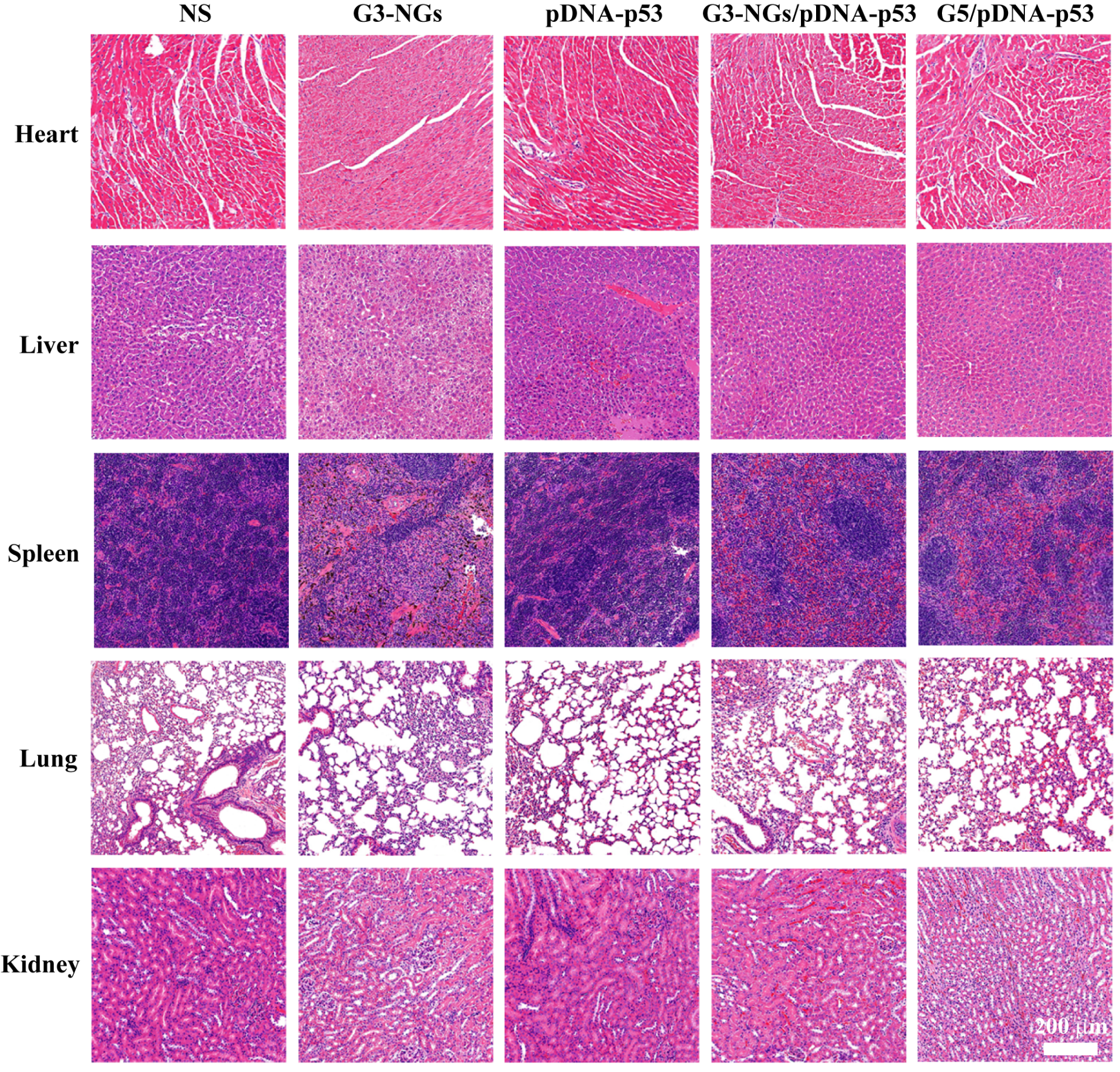


**Figure S21.** H&E staining of heart, liver, spleen, lung and kidney of mice in different groups at day 6 post-injection.

# References

[1] H. Peng, W. J. Xu, A. Pich, *Polym. Chem.* **2016**, *7*, 5011-5022.

[2] L. D. Kong, J. R. Qiu, W. J. Sun, J. Yang, M. W. Shen, L. Wang, X. Y. Shi, *Biomater. Sci.* **2017**, *5*, 258-266.

[3] X. Li, L. D. Kong, W. Hu, C. C. Zhang, A. Pich, X. Y. Shi, X. P. Wang, L. X. Xing, *J. Adv. Res.* **2022**, *37*, 255-266.

[4] J. C. Li, Y. Hu, J. Yang, P. Wei, W. J. Sun, M. W. Shen, G. X. Zhang, X. Y. Shi, *Biomaterials* **2015**, *38*, 10-21.
